# Mechanical polarity links adhesion-regulated protrusions to directional stability in glioblastoma cell migration

**DOI:** 10.1371/journal.pcbi.1014772

**Published:** 2026-09-24

**Authors:** Haruna Tagawa, Daisuke Kanematsu, Asako Katsuma, Naoyuki Inagaki, Yonehiro Kanemura, Yuichi Sakumura

**Affiliations:** 1 Graduate School of Science and Technology, Nara Institute of Science and Technology, Ikoma, Japan; 2 Department of Biomedical Research and Innovation, Institute for Clinical Research, NHO Osaka National Hospital, Osaka, Japan; 3 Data Science Center, Nara Institute of Science and Technology, Ikoma, Japan; University of Massachusetts Amherst, UNITED STATES OF AMERICA

## Abstract

Glioblastoma invasion critically limits therapeutic outcomes, yet the physical principles that govern directional cell migration remain poorly understood. In particular, the degree to which protrusive forces are aligned or cancel each other, and how this governs migration efficiency, have remained unquantified. Here, we introduce mechanical polarity, a quantitative descriptor that captures the alignment of protrusive and adhesive forces driving migration. By integrating time-lapse imaging of the glioblastoma-derived cells with a coarse-grained biophysical model incorporating catch- and slip-bond kinetics, we analyzed motility on fibronectin- and laminin-coated substrates. In our model, the extracellular matrix (ECM) is treated as an external boundary condition that modulates adhesion dynamics, distinct from intrinsic cellular mechanics. We demonstrate that while protrusive activity remains similar across environments, the fibronectin-fitted model exhibited less stable effective adhesion dynamics that led to poorly coordinated protrusions and significant force cancellation, thereby reducing net displacement. These differences are not fully captured by conventional descriptors of protrusion activity but are reflected in mechanical polarity. Conversely, laminin promotes stable adhesions and the alignment of protrusive forces, a state characterized by high mechanical polarity. Our results support mechanical polarity as a candidate physical descriptor linking molecular adhesion kinetics to cell-scale migration stability. This framework provides a quantitative basis for understanding force coordination in glioblastoma cell motility and offers a physical basis for strategies to suppress invasive behavior.

## Author summary

Glioblastoma is an aggressive brain cancer characterized by its ability to infiltrate healthy brain tissue, making complete surgical removal nearly impossible. While it is known that cancer cells move by generating mechanical forces, it has remained unclear how the many small, fluctuating forces produced by cellular protrusions are coordinated to generate stable, directional motion. In this study, we introduce mechanical polarity, a simple measure that quantifies how well these forces are aligned. By combining real-time imaging of patient-derived cancer cells with mathematical modeling, we show that cells can exhibit similar levels of protrusive activity yet move very differently depending on their environment. On some substrates, unstable adhesions cause forces to oppose each other, effectively canceling movement and leading to inefficient, wandering behavior. On others, stable adhesions promote force alignment, enabling efficient and persistent migration. Importantly, these differences are not directly explained by conventional descriptors based on cell shape or activity, but are revealed by mechanical polarity. Our findings provide a new physical framework for understanding how cells organize forces to move and suggest new strategies for limiting invasive behavior by disrupting force coordination.

## Introduction

Tumor cell invasion is a central driver of cancer progression, and deciphering the mechanisms of uncontrolled cell migration remains a critical challenge in tumor biology. Gliomas are the most common primary brain tumors in adults, and more than half are glioblastomas, which are associated with poor patient outcomes [1–4]. The highly invasive motility of glioblastoma cells underlies their resistance to therapy.

Glioblastoma cells extend protrusions known as tumor microtubes [5,6], which dynamically attach to and detach from the extracellular matrix (ECM), thereby regulating migration efficiency and directional stability. However, existing frameworks fail to explain how fluctuating protrusive and adhesive forces are coordinated to produce stable directional migration. To address this problem from a physical perspective, we treat the ECM as an external boundary condition that modulates adhesion dynamics.

Cell migration emerges from the integration of morphological polarization, cytoskeletal remodeling, and adhesion dynamics. Traditionally, directional stability has been attributed to morphological asymmetry [7–10]. However, accumulating evidence suggests that cells can maintain persistent migration even when morphological polarity is weak or misaligned with the direction of movement [11,12], indicating that shape asymmetry alone cannot account for directional persistence.

These observations point to a missing physical link between cell morphology and persistent migration. In particular, the degree to which protrusive forces are aligned or cancel each other, and how this governs directional stability, remains unquantified. Here, we introduce mechanical polarity as a quantitative descriptor of the alignment of protrusive and adhesive forces that underlie directional migration. Unlike morphological metrics, mechanical polarity explicitly captures the coordination of force-generating units.

While many mathematical models describe cells as continuous bodies without explicitly resolving protrusive dynamics [13,14], some frameworks have incorporated force-generating protrusions or protrusion dynamics regulated by intracellular polarity signaling. For example, previous work has shown that coupling polarity signaling, lamellipodial competition, and adhesion feedback can generate distinct migratory phenotypes [15], whereas more recent glioblastoma-specific studies have applied motor-clutch-based frameworks to relate physical migration parameters to glioblastoma cell behavior [16,17].

These studies highlight complementary perspectives, one centered on intracellular signaling-mediated polarity and the other on detailed force transmission through adhesion mechanics. Here, we take an intermediate coarse-grained approach that does not explicitly model full signaling networks or molecular clutch machinery, but instead focuses on protrusion-level force generation and effective force-dependent adhesion kinetics to quantify how force coordination governs migration. This intermediate level of abstraction enables us to link molecular-scale adhesion kinetics to cell-scale migration.

To test this framework, we combined time-lapse imaging of the patient-derived glioblastoma cell line GDC40 [18] under serum-free conditions with simulations of cell migration on fibronectin- and laminin-coated substrates. This design allowed us to disentangle cell-intrinsic mechanical properties from ECM-dependent effects by comparing motility across distinct environments. Our model incorporates force-dependent adhesion kinetics, including catch- and slip-bond behaviors [19–21], and recapitulates ECM-dependent differences in migration.

We show that although protrusive activity remains similar across conditions, fibronectin induces unstable adhesion dynamics that lead to poorly coordinated protrusions and force cancellation, resulting in reduced migration efficiency. In contrast, laminin promotes stable adhesions and alignment of protrusive forces, enabling persistent migration. These differences are not fully captured by conventional descriptors of protrusion activity but are reflected in mechanical polarity.

Together, our results support mechanical polarity as a candidate physical principle linking adhesion dynamics to directional stability in cell migration.

## Results

### Glioblastoma cell migration depends on ECM coating conditions

**Fig 1:**
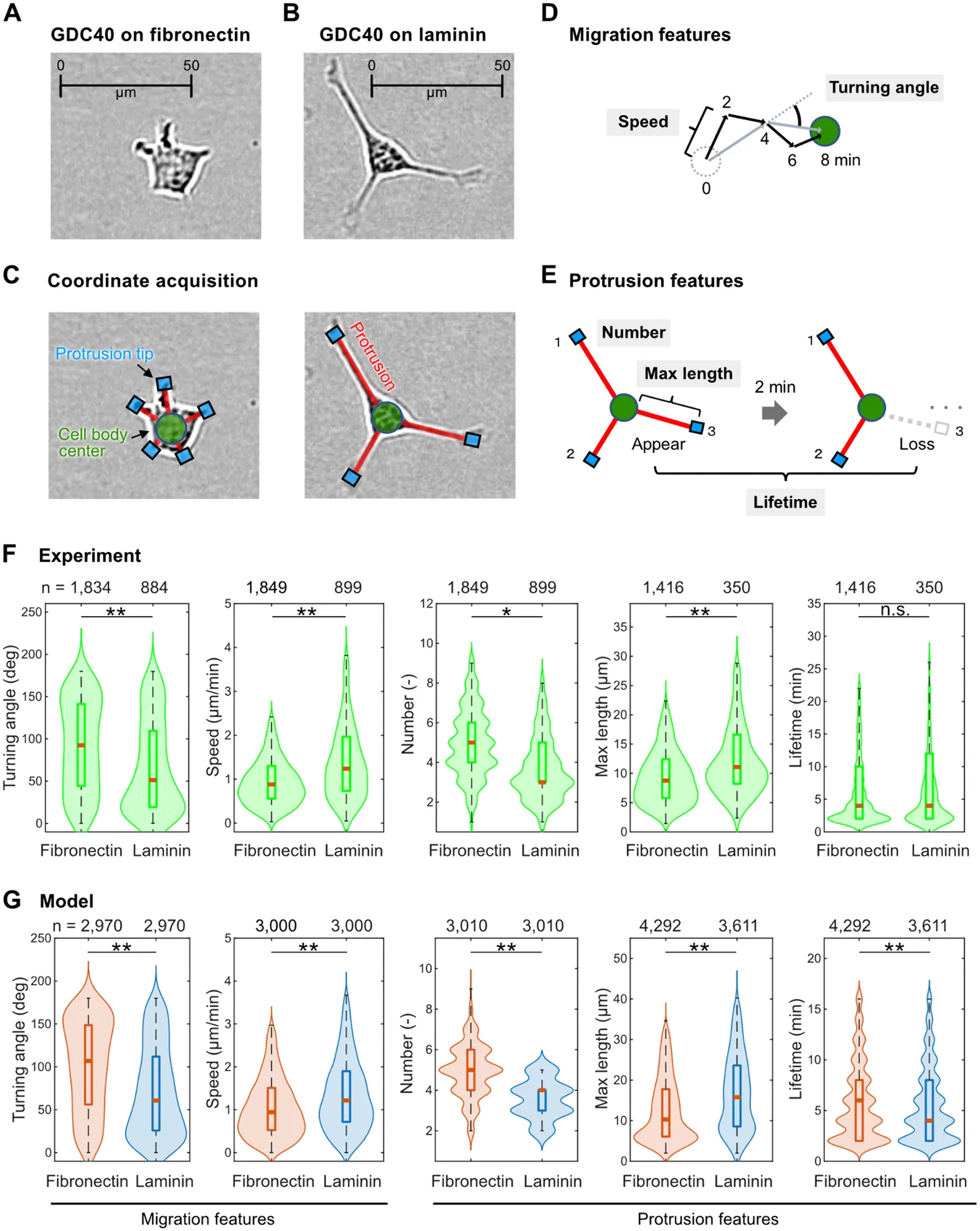
Quantitative features of GDC40 cell migration and protrusion dynamics. (A, B) Phase-contrast images of GDC40 cells on fibronectin and laminin, respectively. (C) Quantified components: cell body center (green), protrusion tips (cyan squares), and protrusion lengths (red lines). (D) Definitions of migration features. Speed was defined as the displacement of the cell body center over 2-minute intervals, whereas the turning angle was defined as the change in migration angle over 4-minute intervals to reduce the effect of short-term fluctuations. (E) Protrusion features include the number per frame, maximum length, and lifetime. (F, G) Quantification results of migration and protrusion features for GDC40 cells on fibronectin (F-GDC40) or laminin (L-GDC40). (F) shows results obtained from actual cell images, while (G) shows results from mathematical model simulations. The sample size n is indicated above each plot. The red line in the box represents the median. Values more than 1.5 times the interquartile range away from the top and bottom of the box were considered outliers. The outliers were omitted from the plots for visibility, but all data were included in the statistical tests and analyses. Statistical differences were assessed using mixed-effects models with ECM condition as a fixed effect and cell identity as a random effect (**p < 0.01; *p < 0.05; n.s., not significant).

We analyzed time-lapse images of GDC40 cells on glass coated with fibronectin (F-GDC40) (Fig 1A, S1 Movie) or laminin (L-GDC40) (Fig 1B, S2 Movie). From the images, we extracted the coordinates of the cell body center and protrusion tips (Fig 1C), and quantified two groups of features: migration (turning angle and speed; Fig 1D) and protrusion features (number, maximum length, and lifetime; Fig 1E).

F-GDC40 frequently changed direction by more than 90° (Fig 1F). In contrast, L-GDC40 mostly turned by less than 90°, indicating higher directional persistence. L-GDC40 showed greater migration speed, longer protrusions, and fewer protrusions than F-GDC40. No significant difference was found in protrusion lifetime. Since cell migration depends on protrusion dynamics [10,22], these results suggest that the differences in protrusion features that we observed can substantially affect migration features.

### Development of a quantitative model of cell migration

Protrusion dynamics are mechanically regulated by adhesion and detachment events at the protrusion tip, as well as changes in tensile forces [23]. We hypothesized that the differences in the mechanical properties of adhesion could alter protrusion number and length, thereby shaping the migration phenotype. To test this, we developed a quantitative physical model based on our previously reported mathematical model of neuronal polarization [24] and conducted simulations.

**Fig 2:**
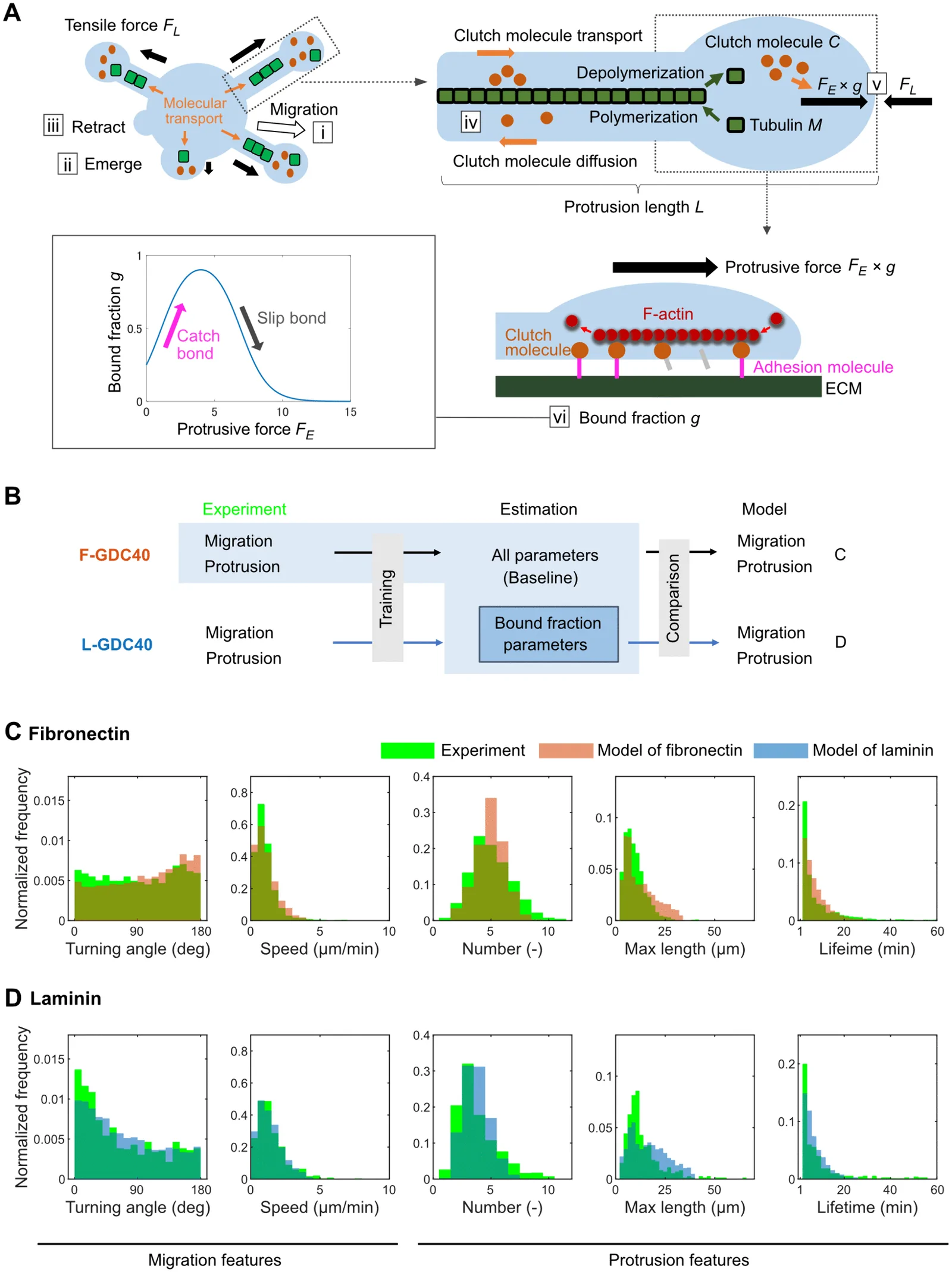
Model construction, parameter fitting, and reproduction of GDC40 cell motility. (A) Schematic of the cell migration model: (i) whole-cell migration driven by protrusion forces; (ii-iii) stochastic emergence and removal of protrusions; (iv) transport of cytoskeletal components (tubulin) and clutch molecules; (v) force balance at the protrusion tip between polymerization-induced protrusive force $F_{E}\;g$ and tension $F_{L}$; (vi) force-dependent bound fraction described by the function $g(F_{E})$, capturing both catch and slip bonds of adhesion molecules. (B) Parameter fitting scheme: All free parameters were first fitted to F-GDC40 data (baseline parameters), followed by selective fitting of the function $g(F_{E})$ to L-GDC40 data. (C, D) Comparison between experimental (green) and simulated (orange or blue) distributions of cell behavior (C: F-GDC40; D: L-GDC40). Sample sizes are shown in Fig 1F **and** 1G.

The model represents a glioblastoma cell as a central cell body with multiple protrusions (Fig 2A). The cell body migrates according to the vector sum of forces generated by the protrusions (**i**). The timing of protrusion emergence is determined by a probability that depends on the remaining amount of cytoskeletal components **(ii)**, and protrusions are removed when their length falls to or below a set threshold (2 μm) **(iii)**. Clutch molecules reversibly link adhesion molecules to the actin cytoskeleton; when engaged, they couple retrograde actin flow to the ECM to generate traction and, by reaction, protrusive force, whereas disengagement releases these forces [25–27]. We therefore hypothesized that protrusion elongation depends on the transport of clutch molecules from the cell body to the tip and their return by diffusion as observed in neuronal cells [28] (**iv**), as well as on the balance between protrusive force and tension at the tip (**v**). The effectiveness of the adhesion is represented by the bound fraction g, defined as the ratio of the number of adhesion molecules bound to the ECM to the total adhesion molecules at the protrusion tip, modeled as a function of protrusive force (**vi**).

At the protrusion level, protrusion length affects the accumulation of clutch molecules, C, at the protrusion tip. This process is represented by the Fick’s-law-based diffusion term in dC/dt (Eq. 10). As protrusions become longer, diffusive redistribution from the protrusion tip toward the cell body becomes slower, thereby promoting the accumulation of C at the tip. The accumulated clutch molecules enhance the generation of protrusive force ($F_{E}$; Eq. 6), and this force feeds back to protrusion elongation through the second term in the equation for $v_{tip}$ (Eq. 4). On the other hand, when the protrusive force $F_{E}$ becomes large, adhesive bonds are more likely to detach, leading to a decrease in the bound fraction (g; Eq. 7). In addition, when protrusions become sufficiently long, the tension $F_{L}$ (Eq. 5) increases, and this force also acts through $v_{tip}$ (Eq. 4) to promote protrusion retraction. Thus, protrusion length, clutch molecule accumulation, protrusive force, adhesion detachment, and tension together form feedback loops that regulate protrusion elongation and retraction.

At the whole-cell level, multiple protrusions compete for limited cytoskeletal resources (M; Eq. 9) and clutch molecules C. Therefore, frequent protrusion emergence distributes the available resources among many protrusions, leading to shorter and less stable protrusions. In contrast, the maintenance of fewer stable protrusions promotes spatially biased force generation, mechanical polarity, and persistent cell migration. Further details of the model and the equations are provided in the Materials and Methods.

### ECM-dependent migration differences reproduced by adhesion mechanics

To validate the model structure, we conducted parameter estimation and simulations (Fig 2B). We first obtained the feature distributions of F-GDC40 (green distributions of Fig 2C), then estimated all free parameters from the distributions and simulated cell migration. We designated this parameter set as the “baseline” (F-ECM condition). Next, using the L-GDC40 feature distributions (green distributions of Fig 2D), we re-estimated only the four adhesion-related parameters ($a_{s},b_{s},\;a_{c},$ and $b_{c}$), keeping the rest fixed (L-ECM condition) (Fig 2B). The details of the estimated adhesion-related parameters will be discussed in the next section.

**Fig 3:**
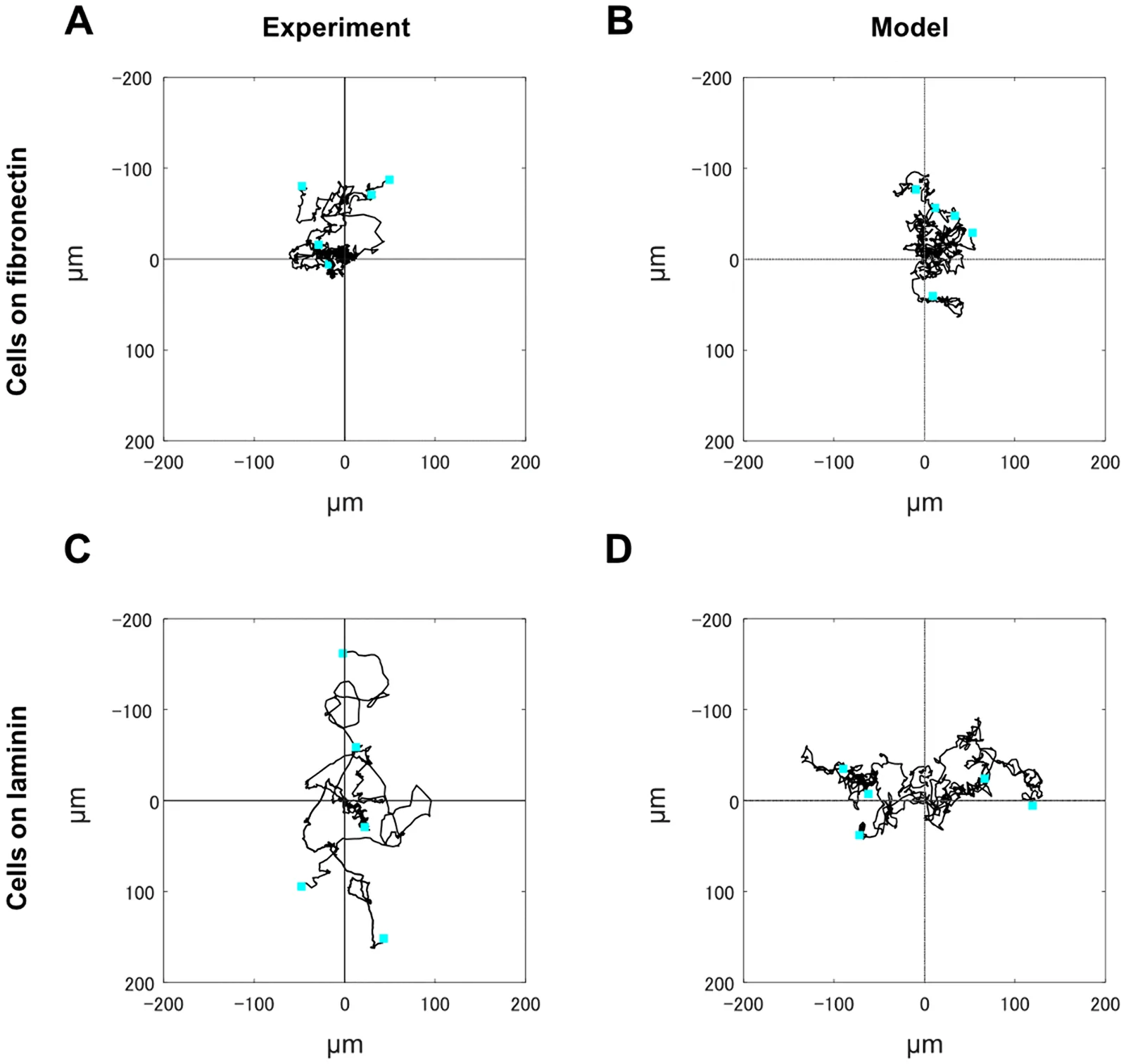
Cell migration trajectories. (A) Trajectories of cells migrating on fibronectin. (B) Simulated migration trajectories using parameters estimated from cell images on fibronectin (F-ECM). (C) Trajectories of cells migrating on laminin. (D) Simulated migration trajectories using parameters $a_{s},b_{s},\;a_{c}$ and $b_{c}$ estimated from cell images on laminin (L-ECM). Each panel shows data from five individual cells.

The model reproduced ECM-dependent differences in migration and protrusion, including a lower turning angle and higher speed in L-GDC40, as well as more numerous and shorter protrusions in F-GDC40 (Figs 1G, 2C, 2D, 3, and S3 and S4 Movies). However, protrusion lifetime was an exception: the model showed a statistically significant ECM-dependent difference in lifetime, whereas the experimental data did not.

We calculated the Jensen–Shannon (JS) divergence to compare model feature distributions with experimental data under each ECM condition. A smaller JS divergence indicates greater similarity between distributions. In both ECM conditions, except for lifetime (which showed no significant differences in the experiments; Fig 1F), the JS divergence was smaller for model–experiment pairs with the same ECM type than for non-matching pairs (Table A in S1 Appendix), indicating that each fitted model generally showed closer agreement with the experimental distributions from its corresponding ECM condition than with those from the other condition. Furthermore, even when adhesion parameters were estimated using only migration features or only protrusion features, the differences in motility between L-GDC40 and F-GDC40 were still successfully reproduced (Fig A and Table B in S1 Appendix).

Notably, these migration features were reproduced solely by modifying adhesion-related parameters. These results suggest that the differences in migratory behavior between F-GDC40 and L-GDC40, as observed in our experiments, can be explained by mechanical differences in adhesion. Moreover, the fact that these distinct migratory patterns emerged by varying only adhesion-related parameters supports the utility of the model for describing how effective adhesion mechanics can generate ECM-dependent migration patterns.

However, alternative parameter combinations might also reproduce the observed ECM-dependent differences in motility. To examine whether the fitted parameter set was unique and whether the model predictions were robust across alternative parameter estimates, we performed 16 independent fitting runs starting from the same initial parameter values (Text E in S1 Appendix). The runs converged to distinct parameter combinations, suggesting that the fitted parameters were not uniquely identifiable (Fig B in S1 Appendix). We also examined correlations among the fitted parameters across the 16 solutions and found that several parameters covaried, suggesting compensatory relationships among them (Fig C in S1 Appendix).

We next examined whether this variability affected the qualitative model predictions. All 16 solutions reproduced the ECM-dependent differences in at least three of the four features that showed significant differences between the fibronectin and laminin conditions in the experimental data (turning angle, migration speed, protrusion number, and maximum protrusion length), and 7 solutions reproduced all four (Table H in S1 Appendix). In addition, the matched model–experiment ECM comparisons showed better agreement than the mismatched comparisons in most fitted solutions (16/16 for the fibronectin-fitted models, 10/16 for the laminin-fitted models). Together, these results suggest that the qualitative conclusion regarding ECM-dependent differences in effective adhesion mechanics was robust across the fitted parameter sets and did not depend on a single solution.

### Differences in adhesion properties alter mechanical interference between protrusions and modulate cell motility

Our model assumes a causal relationship in which the bound fraction of adhesion molecules regulates protrusion features, which in turn influence migration features. To test this assumption, we analyzed the simulation results to quantify how differences in bound fraction and in the resulting tensile forces between the F-ECM and L-ECM conditions contribute to cell migration.

The estimated ratios of the four adhesion-related parameters ($a_{s},b_{s},a_{c},b_{c}$) between the F-ECM and L-ECM conditions were 1.07, 1.38, 0.92, and 1.17, respectively. $a_{s},b_{s},\;a_{c},$ and $b_{c}\;$are composite parameters incorporating the number of adhesion molecules and adhesion sites on the extracellular matrix, bond strength, reaction rate constants, and the length per unit of the cytoskeletal components (Text C in S1 Appendix). $a_{s}$ and $b_{s}$ are attributable to slip bonds, while $a_{c}$ and $b_{c}$ are attributable to catch bonds. $a_{s}$ and $a_{c}$ act as sensitivity parameters, and $b_{s}$ and $b_{c}$ serve as scale parameters (see Methods, Eq. 7).

**Fig 4:**
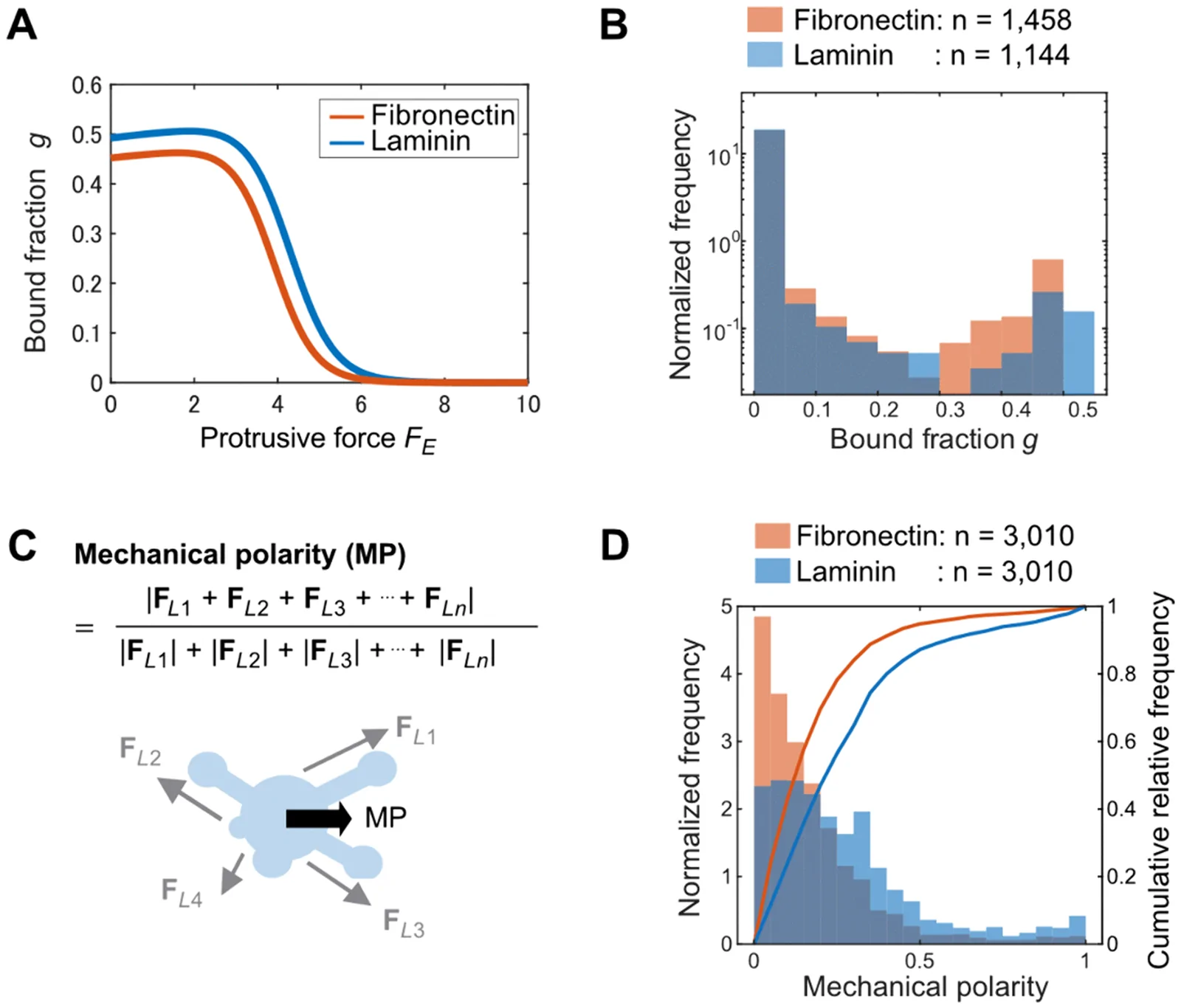
ECM-dependent adhesion dynamics and protrusion force efficiency in the model. (A) The bound fractions $g(F_{E})$ under two ECM conditions. The orange curve represents the baseline parameters estimated from F-GDC40 (F-ECM condition), and the blue curve shows the result of refitting the bound fraction parameters ($a_{c},b_{c},a_{s},b_{s}$) using L-GDC40 data (L-ECM condition). $F_{E}$ denotes the protrusive force excluding the effect of the bound fraction. (B) Log-scaled histograms of bound fraction g measured every 2 minutes for each protrusion in a single simulated cell. Orange and blue represent F-ECM and L-ECM conditions, respectively. (C) Definition of mechanical polarity as the ratio between the net tensile-force vector and the total magnitude of all protrusion forces. (D) Distributions and cumulative plots of mechanical polarity obtained from simulations. Values were sampled every 2 minutes over 10 simulated cells per condition. Data points where protrusions were absent or forces were zero were excluded.

In the F-ECM condition, the lower value of the catch bond sensitivity parameter $a_{c}$ and the higher values of the scale parameters $b_{s}$ and $b_{c}$ indicate that the bound fraction responds more gradually to force and reaches a lower maximum level compared to the L-ECM condition. Consistently, the bound fraction function $g(F_{E}$), computed from these parameters, showed higher values across all force levels $F_{E}$ in the L-ECM condition than in the F-ECM condition (Fig 4A). Notably, the peak value of $g(F_{E}$) was approximately 0.45 for F-ECM and 0.5 for L-ECM, suggesting that cell–ECM adhesion is more unstable and prone to slippage under the F-ECM condition. Because the bound fraction function exhibits a switch-like shape, the distribution of bound fraction values during model simulations was bimodal under both ECM conditions; the L-ECM condition showed a slightly broader distribution (Fig 4B).

To understand how protrusion-generated forces contribute to cell migration, we introduce the concept of mechanical polarity, which represents the forces acting on the cell body that do not completely cancel out but remain biased in a particular direction, thereby driving the displacement of the cell body. Mechanical polarity is defined as the magnitude of the resultant force divided by the sum of the magnitudes of the individual forces (Fig 4C). It quantifies the efficiency of such force generation. A higher value indicates better alignment of forces and more efficient translation of protrusion force into net displacement of the cell body. Polarity, in its original sense, refers to directionality. In this study, polarity is distinguished from biological polarity based on morphological or molecular distributions; instead, it is defined as the directional property arising from non-zero physical forces acting on the cell body.

Simulation results for mechanical polarity are shown in Fig 4D. Values below 0.2 occurred more frequently under F-ECM conditions, while values above 0.2 were more common under L-ECM. The statistical test (the mixed-effects model with ECM condition as a fixed effect and cell identity as a random effect) revealed a significant difference in the values between F-ECM and L-ECM (p < 0.01). This indicates that in L-GDC40, which exhibit higher speed and directional persistence, protrusion forces are less likely to cancel out and more efficiently drive directional migration.

We further analyzed the simulated trajectories to quantitatively evaluate the relationships between mechanical polarity and dynamic migration features under each condition. Specifically, along the same simulated trajectories, mechanical polarity and the corresponding speed and turning angle were calculated at 2-minute intervals, and their correlations were evaluated using Spearman’s correlation coefficient. As a result, mechanical polarity showed moderate positive correlations with speed (r = 0.4 to 0.6) and weak negative correlations with turning angle (r = −0.3 to −0.1) (Figs D and E in S1 Appendix). The weaker association with turning angle may reflect the fact that turning angle depends on temporal changes in migration direction, whereas mechanical polarity was evaluated as an instantaneous force-alignment measure. These results support our interpretation that mechanical polarity captures the efficiency of coordinated force generation during migration, although its relationship with short-timescale directional changes is weaker.

### Protrusion frequency regulates migration efficiency and offers a potential strategy for motility suppression

Based on our prior results, we hypothesized that modulating protrusion features, while keeping adhesion parameters constant, could control mechanical polarity and thereby influence migration features. In particular, converting the “few long protrusions” characteristic of L-GDC40 into the “many short protrusions” observed in F-GDC40 may reduce motility.

**Fig 5:**
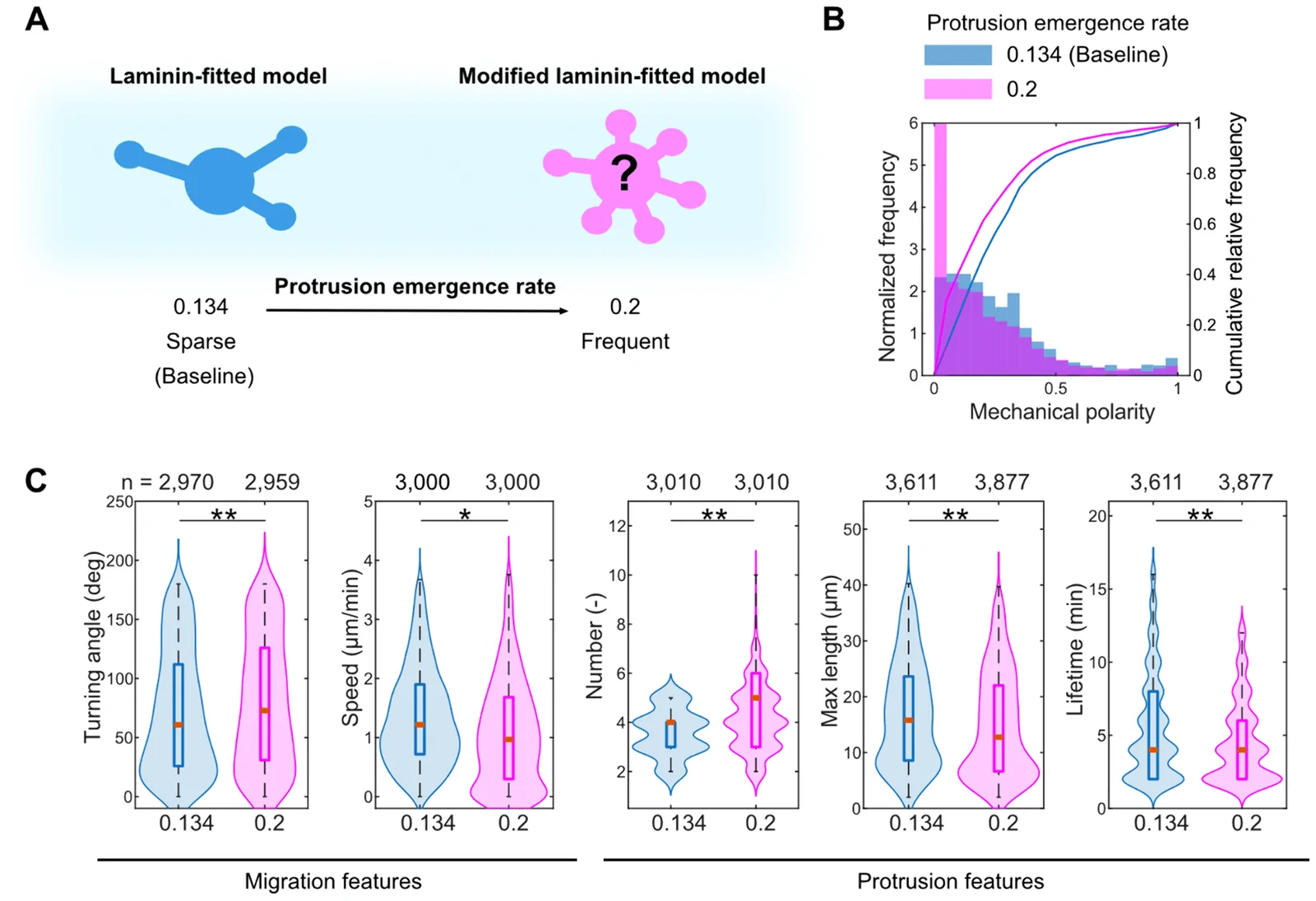
Effect of increased protrusion emergence rate on cell motility. (A) Comparison of motility under two different values of the protrusion emergence rate parameter $k_{pr}$: baseline ($k_{pr}=\text{0}.\text{1}\text{3}\text{4}$) and elevated ($k_{pr}=\text{0}.\text{2}$). Parameters other than $k_{pr}$ were set to the values under L-ECM conditions. (B-C) Comparison of motility features at $k_{pr}=\text{0}.\text{1}\text{3}\text{4}$ (blue) and $k_{pr}=\text{0}.\text{2}$ (magenta). Statistical differences were assessed using mixed-effects models with protrusion emergence rate as a fixed effect and cell identity as a random effect (*p < 0.05; **p < 0.01). The sample size n is indicated above each plot. The red line in the box represents the median. Values more than 1.5 times the interquartile range away from the top and bottom of the box were considered outliers. Outliers were omitted from the box plots for visibility, but all data were included in the statistical tests and analyses.

To test this hypothesis, we increased the protrusion emergence rate parameter $k_{pr}$ from its estimated value of 0.134 to 0.2, while keeping all other parameters fixed under L-ECM conditions, and conducted simulations for 10 cells over 600 minutes (Fig 5A). Results for mechanical polarity, turning angle and speed of migration, number, maximum length, and lifetime of protrusions at $k_{pr}=\text{0}.\text{2}$ are shown in Fig 5B and 5C and Table C in S1 Appendix. As a result of the statistical tests (the mixed-effects models with protrusion emergence rate as a fixed effect and cell identity as a random effect), mechanical polarity decreased (p < 0.05). The migration speed decreased. Some cells in the simulation stopped moving, and the frequency of zero-speed events increased. A significant difference was observed in the turning angle. The number of protrusions increased beyond the levels observed under the fibronectin conditions, and the length decreased. Protrusion lifetimes also slightly decreased. We further varied $k_{pr}$ from 0.1 to 0.2 in increments of 0.02, conducting simulations of 10 cells over 600 minutes for each condition, with data recorded every 2 minutes. The trends were largely similar (Fig F in S1 Appendix).

Since protrusions serve as the primary force generators for cell migration [29], the observed decrease in motility can be attributed to changes in protrusion features, specifically the increase in number and reduction in length. Collectively, these results suggest that both the protrusions and their mechanical balance play critical roles in regulating the efficiency of glioblastoma cell migration.

## Discussion

We show that directional migration in glioblastoma cells is linked by the coordination of protrusive forces rather than by protrusion activity or morphology alone. By combining experiments and modeling, we demonstrate that ECM-dependent adhesion dynamics regulate this coordination, giving rise to differences in migration efficiency and persistence. This principle is captured by mechanical polarity, which quantifies the alignment of force-generating protrusions.

Our findings indicate that ECM composition modulates migration by altering effective adhesion dynamics at protrusion tips. Under laminin conditions, cells exhibit fewer but longer protrusions, with higher directional persistence and speed. In contrast, fibronectin leads to more frequent, shorter protrusions and inefficient, less persistent migration. These ECM-dependent differences in protrusion morphology and dynamics are captured in our experimental observations (Fig 1F), and our model suggests they arise from changes in adhesion stability (Fig 4A and 4B), which in turn affect how protrusive forces are coordinated across the cell (Fig 4C and 4D).

Importantly, our model provides a framework to infer how adhesion-dependent force coordination gives rise to migration behavior, rather than merely reproducing observed dynamics. By focusing on adhesion-related parameters while keeping other components fixed, we show that within the present model, variation in adhesion-related parameters alone was sufficient to reproduce the observed ECM-dependent differences in migration. This supports the idea that physical adhesion properties act as key regulators of cell motility.

Traditionally, ECM-dependent differences in migration have been attributed primarily to intracellular signaling pathways [8,30–32]. In contrast, our framework does not explicitly model these biochemical processes; instead, their net effects are captured through effective adhesion parameters that shape force coordination across protrusions. This abstraction allows us to connect molecular-scale interactions to cell-scale behavior in a tractable manner.

Note that, in our framework, ECM-dependent differences are represented primarily through the stability of the effective bound fraction. Once adhesions become more stable, protrusions are maintained and extended more easily, and clutch molecules are more likely to remain near protrusion tips. Accordingly, the apparent suppression of retrograde diffusion toward the cell body is interpreted as a consequence of adhesion stabilization, rather than its primary cause.

A central implication of our study is that the direction of cell body movement is determined by the vector sum of forces generated by multiple protrusions over time. We define this coordination, representing the net bias of force generation, as mechanical polarity. Mechanical polarity complements, rather than replaces, morphological polarity, providing directional stability even when morphological asymmetry is weak or transient.

These results are consistent with previous studies showing that ECM adhesion influences cell behavior. For example, neuronal axon outgrowth is guided by differences in adhesion strength [33], and macrophages exhibit ECM-dependent changes in migration mode associated with adhesion engagement [34]. Our work extends these findings by providing a quantitative framework that links adhesion dynamics to force coordination and migration outcomes.

At the molecular level, differences in adhesion dynamics between laminin and fibronectin likely arise from distinct ligand–receptor interactions. GDC40 cells express L1CAM, which has been reported to exhibit catch bond–like binding to laminin [35], and increased L1CAM expression enhances migratory capacity [36]. These interactions may stabilize adhesions under load via catch-bond kinetics, preventing premature detachment and allowing protrusions to mature. This stabilization promotes efficient force transmission and alignment, thereby increasing mechanical polarity.

In contrast, weaker or more transient adhesion under the fibronectin conditions may reduce the bound fraction under force, leading to shorter protrusions that fail to accumulate resources for sustained elongation. As a result, multiple weak protrusions emerge, generating forces in diverse directions that cancel each other and reduce net migration efficiency. These molecular differences can thus be interpreted as mechanisms that modulate mechanical polarity by altering force transmission and coordination.

Although integrins also mediate adhesion to ECM ligands and exhibit force-dependent binding behavior [20,27], their contribution to ECM-specific differences may be distinct from that of L1CAM. Integrins bind to both fibronectin and laminin, whereas L1CAM interactions appear more specific, suggesting that multiple adhesion systems may differentially regulate force coordination. Future quantitative measurements of these interactions will be necessary to refine this interpretation.

Although the fitted differences in the adhesion-related parameters between the two ECM conditions were modest, their effects were amplified through temporal accumulation in the model dynamics. At each time step, these parameters altered the stability of protrusion–substrate adhesion and thereby shifted the balance between protrusion extension, maintenance, and retraction. Repetition of these small differences over time progressively changed protrusion organization and the degree of force coordination across the cell. Consequently, even modest differences in effective adhesion properties could lead to substantial differences in emergent migration behavior, including migration speed and directional persistence.

**Fig 6:**
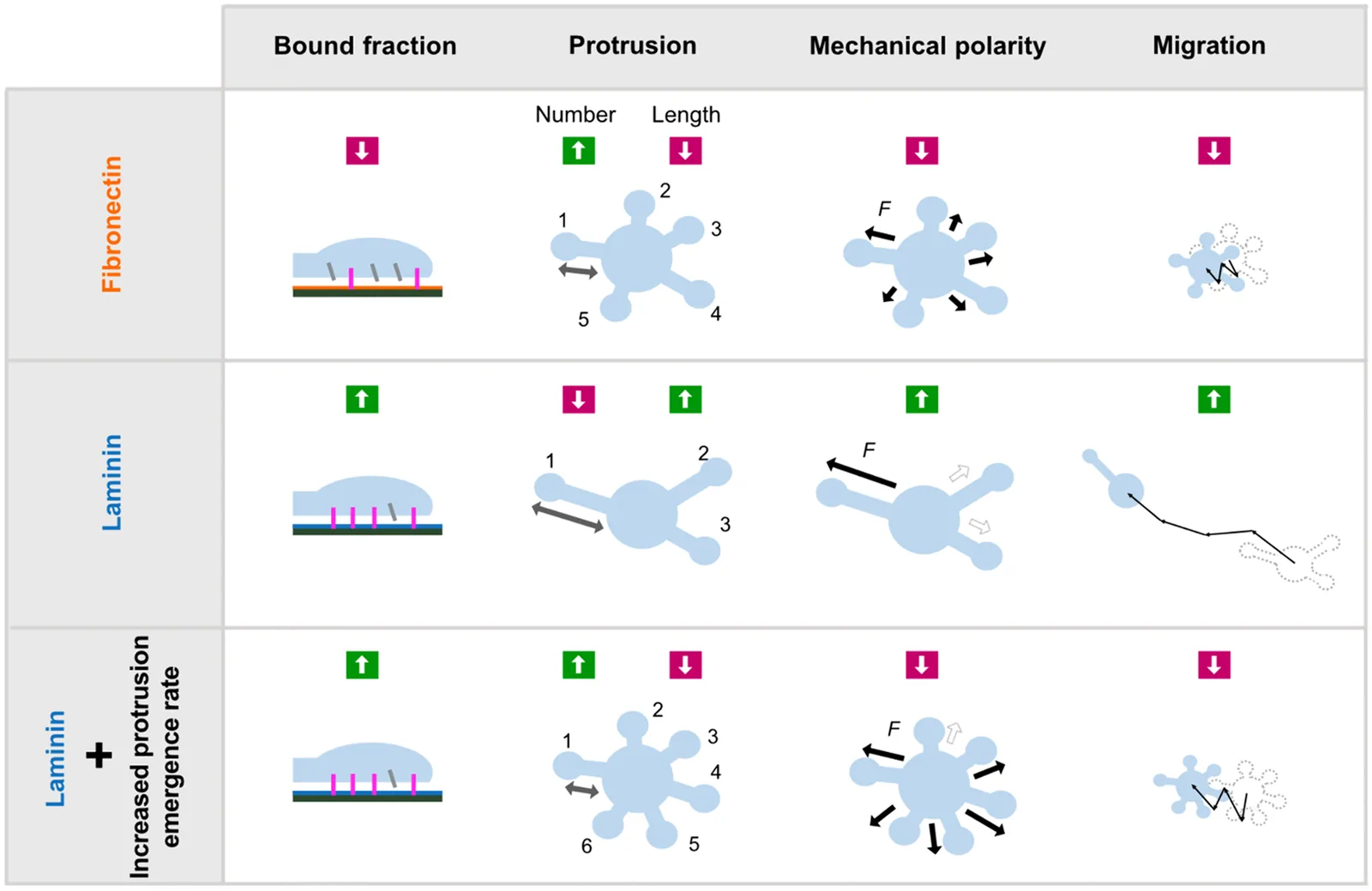
Conceptual model of how ECM and protrusion dynamics shape cell migration. Summary of migration-related features under three conditions: fibronectin (top), laminin (middle), and laminin with increased protrusion emergence rate (bottom). Key characteristics (bound fraction, protrusion morphology, mechanical polarity, and overall migration) are represented schematically. In fibronectin conditions, weak adhesion leads to numerous short protrusions, resulting in force cancellation and impaired migration. In laminin, stronger adhesion with fewer, longer protrusions enables better force alignment and efficient migration. When the protrusion emergence rate is artificially increased under laminin conditions, the system shifts toward reduced mechanical polarity and diminished migration. A higher protrusion emergence rate produces more protrusions, but also increases competition among them for limited protrusion-supporting resources. This reduces the stability of individual protrusions and leads to a decrease in average protrusion length.

Finally, our simulations show that increasing the protrusion emergence rate alone can disrupt migration efficiency. In the model, a higher protrusion emergence rate increases the number of newly formed protrusions, but does not increase the total amount of resources available to sustain them. Thus, increasing protrusion initiation can reduce average protrusion length. As protrusion number increases and the length becomes shorter, force interference becomes more pronounced, reducing net force alignment (Fig 6). This finding suggests that excessive protrusion formation may impair mechanical polarity and suppress directional migration. Experimental manipulation of regulators such as GAP-43 [5] and Ttyh1 [37,38] may provide a means to test these predictions.

Together, our results support mechanical polarity as a candidate physical principle linking adhesion dynamics to directional stability in glioblastoma cell migration. This framework provides a basis for understanding how GDC40 cells organize forces to move and provides experimentally testable hypotheses for examining whether modulation of adhesion molecules such as L1CAM can alter force coordination and migration behavior.

### Future directions

Future extensions of this framework should incorporate heterogeneity in adhesion dynamics across individual protrusions. In the current model, adhesion properties are assumed to be uniform; however, in reality, each protrusion likely experiences distinct molecular compositions and mechanical environments. Incorporating such heterogeneity in adhesion parameters would enable a more accurate representation of how local variations influence force coordination and, consequently, mechanical polarity. Furthermore, the observed variability in protrusion number, length, and lifetime (Fig 2C and 2D) may be better captured by incorporating stochastic fluctuations in molecular components, such as molecular clutch proteins or cytoskeletal regulators.

One limitation of the current model is that it predicts an ECM-dependent difference in protrusion lifetime, whereas no significant difference was detected in the experimental data. This discrepancy may partly reflect the limited experimental sample size. However, the ECM-dependent difference in protrusion lifetime remained statistically significant in all 100 subsampling iterations, in each of which 5 of the 10 simulated cells were randomly selected from each ECM condition (Text D, Table I, and Fig G in S1 Appendix). This suggests that the discrepancy is not explained solely by the difference in cell number between the simulations and experiments, and that the present coarse-grained formulation may overestimate the extent to which adhesion-dependent effects are translated into protrusion lifetime differences. Thus, protrusion lifetime appears to be the feature least well captured by the current model and may require additional mechanisms in future refinements.

Furthermore, although the mixed-effects analysis accounts for repeated measurements within individual cells, the limited number of independent cells remains a limitation and may restrict the generalizability of the experimental findings. Future studies with larger numbers of cells and a broader range of cell types would help further assess the robustness and generality of the proposed mechanism.

Another crucial direction is to integrate active contractile feedback into the model. Myosin II-mediated contractility is a key driver of glioma invasion [39], and force-dependent recruitment of myosin II has been reported in other migratory systems [40]. These findings suggest that local tension may enhance myosin II accumulation and contractility, potentially forming a feedback loop that modulates adhesion dynamics. Incorporating such mechanisms, for example by coupling adhesion kinetics to local myosin II activity, would provide deeper insight into how mechanical polarity is dynamically regulated under varying mechanical loads.

An additional point that emerged from the temporal-resolution analysis is that the current model does not fully capture short-timescale directional fluctuations, particularly under the fibronectin condition. When turning angle was evaluated over 2-minute intervals, the experimental distributions remained similar to those obtained at 4-minute intervals (Fig 2C, panel C of Fig H in S1 Appendix; green histogram), whereas the simulated fibronectin cells showed an excess of small turning angles (panel C of Fig H in S1 Appendix; orange histogram). This suggests that the model produces smoother short-timescale trajectories than those observed in real cells. One possible interpretation is that the present coarse-grained formulation underrepresents rapid fluctuations in migration direction arising from local protrusion dynamics, transient force imbalance, or other stochastic processes that affect real cells on short timescales. In addition, small errors in cell-center estimation may contribute to short-timescale variability in experimental trajectories. Considering such fast fluctuations more explicitly may improve agreement between simulations and experiments at higher temporal resolution. Importantly, this limitation does not alter the main conclusion of the study, because the ECM-dependent difference in directional stability was consistently observed in the experiments and the model at the 2-minute timescale (panels A and B of Fig H in S1 Appendix).

More broadly, the framework presented here offers a physics-based, quantitative basis for predicting how changes in molecular-scale adhesion and protrusion dynamics govern macro-scale migration. Its simplicity and extensibility make it suitable for systematic exploration of parameter spaces and for generating experimentally testable predictions. In particular, our model predicts that perturbations disrupting force coordination will reduce mechanical polarity and suppress directional migration, suggesting potential strategies for limiting invasive behavior through modulation of force coordination.

Mechanical polarity, as defined in this study, is a model-derived descriptor of the degree to which protrusive and adhesive forces are aligned rather than canceled. Although it was not directly measured here, experimental approximation may be possible. In particular, traction force microscopy (TFM) enables quantitative mapping of cell-generated traction forces and has been widely used to analyze the spatial and temporal organization of forces during cell migration [41–43]. In addition, quantitative analyses of focal adhesion dynamics have shown that adhesion assembly, disassembly, and stability are spatially patterned in migrating cells and are closely linked to traction-force organization [44]. Together, these approaches could provide experimental proxies for the asymmetry and net alignment of protrusive forces, and thus for mechanical polarity, in future studies.

## Materials and methods

### Ethics statement

In this study, we used time-lapse images of the glioblastoma-derived cell line GDC40 in vitro. GDC40 is a cell line isolated and established from clinical samples of a glioblastoma patient [18]. The culture of patient-derived cells for image acquisition was conducted at Osaka National Hospital in accordance with the principles of the Declaration of Helsinki, following approval from the Institutional Review Board of Osaka National Hospital (approval number: 713). Written informed consent was obtained from the patient.

### Experimental procedure

An IncuCyte ImageLock 96-well plate (Sartorius, Goettingen, Germany) was coated with laminin (Sigma; L2020 mouse, 1 mg/mL solution) at 2 μg/cm^2^ or fibronectin (Sigma; F1141 bovine plasma, 0.1% solution) at 5 μg/cm^2^ and incubated at 37 °C for 2 h, washed three times with PBS, and then used for analysis. The dissociated cells were seeded on a laminin- or fibronectin-coated IncuCyte ImageLock 96-well plate at a density of 3 × 10^2^ cells/well and cultured in the IncuCyte ZOOM live imaging system (Sartorius), which contained a 20 × objective lens, at 37 °C in a 5% CO2 incubator. Time-lapse images were obtained every 2 min for 48 h. Image frames were excluded from the analysis during periods when the cell was attached to other cells.

For both laminin and fibronectin conditions, we analyzed five cells each. The spatial conditions of the ECM were uniform, and the conditions of ECM stiffness and cell density were the same. The migration of each cell was recorded using hundreds of time-lapse images. The number of images per cell is listed in Table D in S1 Appendix. Videos of the cells on fibronectin and laminin are shown in S1 and S2 Movies.

### Quantification of glioblastoma cell motility

We obtained coordinates of the cell body center and protrusion tips from time-lapse images of GDC40 on fibronectin (F-GDC40) and GDC40 on laminin (L-GDC40) (Fig 1A-1C). The cell body coordinates were defined as the center of the inscribed circle of the cell body. Protrusions were defined as local outward extensions of the cell contour when the cell body was approximated as a circle. Vertex-like points at angular edges of the cell perimeter were also classified as protrusions (see Fig 1A and 1C). The protrusion tips were visually determined. Using these coordinates, we calculated five features: migration features (Fig 1D), which include migration turning angle and speed, and protrusion features (Fig 1E), which include the number of protrusions at each time point, the maximum length and lifetime of each protrusion. Speed was measured as the displacement of the cell body center over 2-minute intervals. Turning angle was quantified using 4-minute intervals. We used a longer interval for turning angle than speed because directional estimates at shorter intervals are more sensitive to short-timescale fluctuations, including small centroid displacements and rapid local shape changes, which can obscure differences in directional stability at the timescale of interest. We additionally calculated turning-angle distributions using a 2-minute interval and compared the results between experiments and simulations (Fig H in S1 Appendix). Image processing and quantification were performed using MATLAB R2024a (MathWorks, Inc.) (Text A, Figs I and J in S1 Appendix).

### Quantitative modeling of glioblastoma cell migration

#### Model overview.

We constructed a migration model of glioblastoma cells (Fig 2A) based on a previously developed mathematical model of neuronal polarization [24]. In that model, neurons acquire protrusive forces at the tips via clutch molecules, which transmit the force of actin retrograde flow to the ECM. The protrusion length is determined by the mechanical balance between this protrusive force and tension. This mechanism, in which protrusion tips generate traction forces, is also relevant to glioblastoma cell motility [45].

In contrast to the neuronal model, however, the cell body of glioblastoma cells is not fixed but migrates as it is pulled by protrusions. Furthermore, in the neuronal model, the transmission of force via actin flow is regulated by the concentration of clutch molecules, while the force transmitted to the ECM is fixed and does not account for adhesion detachment. In this study, we extended the model by allowing the cell body to migrate in response to protrusive forces and by introducing an ECM-dependent force transmission function that permits detachment (Text B in S1 Appendix). The full mathematical formulation of the model is presented below. Lists of parameters, variables, and expressions used in this model are shown in Tables E-G in S1 Appendix.

#### Dynamics of cell body and protrusion tip.

The glioblastoma cell body migrates by traction of protrusions (panel A of Fig K in S1 Appendix). The protrusions are elongated when their tips bind to the ECM and exert stresses on the cell body (panel B of Fig K in S1 Appendix). Assuming that inertial effects can be ignored because the environment in which the cell migrates is highly viscous, the position vector of the cell body center at time t (${𝐱}_{cb}(t)$) is proportional to the sum of the forces:


$$\begin{array}{c} \frac{d}{dt}{𝐱}_{cb}(t)=\eta \sum_{i}^{n}{𝐅}_{L,\;i}(t), \end{array}$$ (1)

where ${𝐅}_{L,\;i}(t)$ is the vector of tensile force of the *i*-th protrusion, parallel to the relative vector 𝐋(t) from the cell body center to the protrusion tip; η is the motility coefficient. As the cell body migrates according to this equation, the relative direction of each protrusion changes accordingly (Fig L in S1 Appendix), and ${𝐅}_{L,\;i}(t)$ also changes.

From the definition of the vector above, the position vector of the protrusion tip at time t is the sum of ${𝐱}_{cb}(t)$ and the relative vectors 𝐋(t) from ${𝐱}_{cb}(t)$ to the protrusion tip:


$$\begin{array}{c} \begin{array}{c} {𝐱}_{tip}(t)={𝐱}_{cb}(t)+𝐋(t)\;. \end{array} \end{array}$$ (2)

Its velocity has a direction parallel to the protrusion. It can be described as


$$\begin{array}{c} \begin{array}{c} \frac{d}{dt}{𝐱}_{tip}(t)=v_{tip}(t)\frac{𝐋(t)}{\left\|𝐋(t)\right\|}\;, \end{array} \end{array}$$ (3)

(Fig L in S1 Appendix).

#### Model of elongation velocity at protrusion tip.

Protrusions elongate through tubulin polymerization and retract through the disassembly of microtubules. The energy barrier for tubulin polymerization increases due to the load energy imposed by the membrane tension, and the membrane movement velocity is described by a force–velocity relationship [46,47]. Therefore, the membrane velocity at the protrusion tip is


$$\begin{array}{c} \begin{array}{c} v_{tip}=v_{p}\frac{M}{E_{m}}-v_{n}\text{exp}\left(F_{L}-F_{E}\cdot g\left(F_{E}\right)\right). \end{array} \end{array}$$ (4)

Here, $v_{p}$ and $v_{n}$ are polymerization and depolymerization constants, and $E_{m}$ is the total tubulin amount (constant). $F_{L}$ and $F_{E}$ are variables related to the tension and protrusive force, respectively, and the function g represents the binding ratio of the adhesive molecules. For clarity, the protrusion index i and time t are omitted. The first term represents the polymerization rate dependent on the ratio of the time-varying tubulin monomer concentration M to $E_{m}$, and the second term represents the disassembly rate based on the Arrhenius equation, which is force-energy dependent.

$F_{L}$ represents the effect of nonlinear stress on microtubule depolymerization promotion due to protrusion elongation (dimensionless). Based on the previous study [24], this effect can be expressed as a monotonically increasing function of protrusion length L:


$$\begin{array}{c} \begin{array}{c} {\;F}_{L}(L)=\frac{a_{L}\text{ln}(L/L_{\text{0}})}{\text{ln}(K_{L}/L_{\text{0}})+\text{ln}(L/L_{\text{0}})}\;, \end{array} \end{array}$$ (5)

where$\;a_{L}$ is the maximum value,$\;K_{L}$ is the half-value constant, and $L_{\text{0}}$ is the base length. This is a form of the first-order Hill equation applied to a logarithmic function, exhibiting linearity according to Hooke’s law when the protrusion is short. However, the rate of increase in stress decreases sharply as the protrusion lengthens to a certain extent [29].

$F_{E}\;$represents the effect of protrusive force on suppressing microtubule disassembly (dimensionless). It is a Hill-type increasing function of the clutch molecule concentration C [24]:


$$\begin{array}{c} \begin{array}{c} {\;F}_{E}(C)=\frac{a_{E}C^{h_{E}}}{{K_{E}}^{h_{E}}+C^{h_{E}}}, \end{array} \end{array}$$ (6)

where $a_{E}$ is the maximum value, $K_{E}$ is the half-value constant, and $h_{E}$ is the Hill coefficient.

$g\left(F_{E}\right)$ represents the bound fraction of adhesion molecules. We introduced this function into the glioblastoma cell migration model. It expresses the binding ratio of a population of adhesion molecules rather than a single adhesion molecule. The protrusive force by clutch molecules is generated when clutch molecules bind to adhesion molecules such as L1CAM or integrin on the cell membrane. Adhesion molecules on the cell membrane repeatedly form catch bonds and slip bonds with ligands on the ECM [21,48]. In this study, we expressed the force transmitted via the clutch as the product of $g\left(F_{E}\right)$ and $F_{E}$, representing the effective protrusive force effect.

We derived the bound fraction as a function of the clutch-induced protrusive force $F_{E}$ based on previous studies [21,49] (Text C in S1 Appendix):


$$\begin{array}{c} \begin{array}{c} g\left(F_{E}\right)=\frac{\text{1}}{{b_{s}e}^{a_{s}F_{E}}+b_{c}e^{{-a}_{c}F_{E}}\;+\text{1}}\;. \end{array} \end{array}$$ (7)

Here, $a_{s}$ and $b_{s}$ are parameters related to slip bonds, and $a_{c}$ and $b_{c}$ are parameters related to catch bonds. $g\left(F_{E}\right)$ takes values between 0 and 1 (Fig 2A**-vi**). In the denominator, ${b_{s}e}^{a_{s}F_{E}}$ represents the reduction effect due to slip bonds, while $b_{c}e^{{-a}_{c}F_{E}}$ represents the increase effect due to catch bonds. The parameters were assumed to depend on the ECM.

#### Protrusion emergence and removal, and dynamics of cytoskeletal component amounts.

Based on previous studies [50,51], the protrusion emergence was probabilistically modeled. Under the condition that the amount of free tubulin M is greater than or equal to the amount required to form the initial protrusion length $L_{\text{0}}$, the protrusion emergence probability per time step Δt was modeled with a probability of


$$\begin{array}{c} \begin{array}{c} k_{p}(M)=k_{pr}{\left(\frac{M}{E_{m}}\right)}^{\text{4}},\; \end{array} \end{array}$$ (8)

where $k_{pr}$ is a coefficient representing the basic protrusion emergence rate, and $E_{m}$ denotes the total tubulin amount. Therefore, when the free tubulin ratio is small, the emergence probability decreases with the fourth power of the free tubulin ratio [50,51]. The direction of protrusion emergence is randomly determined around the cell body, and the emerged protrusions begin to elongate from the initial length $L_{\text{0}}$. Simultaneously, M decreases by an amount equivalent to $L_{\text{0}}$. Additionally, a protrusion was removed when its length became equal to or less than the initial length $L_{\text{0}}$.

Free tubulin M increases or decreases according to the length $L_{i}$ of each protrusion. When there are n protrusions, M is given by the following equation:


$$\begin{array}{c} M=E_{m}-\sum_{i}^{n}L_{i}\text{.} \end{array}$$ (9)

For clarity in the mathematical formulation, the amount of cytoskeletal components was rescaled to have the same unit as the protrusion length.

#### Clutch molecule transport to protrusion tip and diffusion.

Based on previous studies [24], the dynamics of clutch molecule concentration C at the tip of the protrusion were described as follows:


$$\begin{array}{c} \begin{array}{c} V\frac{dC}{dt}=-\frac{AD}{L}\left(C-C_{\text{0}}\right)+rV_{\text{0}}C_{\text{0}} \end{array}, \end{array}$$ (10)

where constants A, D, V, and $V_{\text{0}}$ represent the cross-sectional area of the protrusion, the diffusion coefficient, the volumes of the protrusion tip and the cell body, respectively, and the remaining symbols are variables or functions. For clarity, the time t and protrusion index i are omitted. $C_{\text{0}}$ is the clutch molecule concentration in the cell body, which changes while preserving the total amount $E_{C}=V_{\text{0}}C_{\text{0}}+\sum_{i}V_{i}C_{i}$.

The first term is a length-dependent diffusion term from the protrusion tip to the cell body based on the one-dimensional Fick’s law [52]. The second term represents active transport from the cell body to the protrusion tip, which is determined by the transport rate r and $C_{\text{0}}$.

### Parameter estimation and numerical simulation

This model contains about six more parameters than the neuronal polarization model due to changes such as introducing a bound fraction in the neural polarization model, resulting in a total of about 18 parameters. The estimation of these parameters was aimed at approximating the behavior of GDC40; both GDC40 and model glioblastoma cells have randomness in their motion and cannot be exactly matched to a specific individual motion. Therefore, the parameters were optimized so that the distributions of the quantitative features for the migration and protrusions of GDC40 (Fig 2C and 2D) were similar to the corresponding distributions in the model. Note that some parameters were determined based on experimental data (Fig M in S1 Appendix) or previous studies (Table E in S1 Appendix).

For this purpose, JS divergence was used as an indicator to evaluate the similarity between distributions (Text D in S1 Appendix), calculated for each of the five features, and the sum of these was used as the objective function:


$$\begin{array}{c} \begin{array}{c} J(\theta )=\sum_{j=\text{1}}^{\text{5}}JS\left(p_{j}(\theta ),q_{j}(\theta )\right),\; \end{array} \end{array}$$ (11)

where $p_{j}(\theta )\;$represents the distribution obtained from simulation results, and $q_{j}(\theta )$ represents the distribution obtained from quantitative results of images, and j corresponds to turning angle, speed, protrusion number, protrusion maximum length, and protrusion lifetime. We chose the JS divergence because the fitting was based on histogram distributions evaluated on a common set of bins, and we sought a symmetric measure of differences in probability mass between experimental and simulated distributions. Unlike the Kullback–Leibler (KL) divergence, the JS divergence is symmetric and remains finite when the two distributions have non-overlapping support, making it more stable for sparsely populated histogram bins. We also considered the Wasserstein distance. However, the Wasserstein distance explicitly incorporates the metric distance between bins and therefore emphasizes how far probability mass must be transported along the variable axis. In the present study, we prioritized differences in the relative probability assigned to the observed histogram bins, particularly for the bounded and potentially multimodal turning-angle distributions.

Parameter estimation was performed using a custom stochastic local search procedure implemented in MATLAB. Starting from an initial parameter set, candidate parameter values were generated by adding Gaussian perturbations to the current parameter values. For each candidate parameter set, the model was simulated and the objective function was evaluated. Candidate parameters were accepted only when they decreased the objective value; otherwise, the previous parameter set was retained. This procedure was repeated until no improvement in the objective value was observed for 500 consecutive proposal steps, and the final retained parameter set was taken as the estimated solution. The standard deviation of the Gaussian perturbation was set to 0.1 or 0.2 times the current value of each parameter. Eight independent runs were performed for each setting, and the parameter set yielding the minimum JS divergence was adopted. The initial parameter values were set as follows: $E_{C}=\text{2}.\text{5},\;\eta =\text{1}.\text{5},\;h_{E}=\text{2},\;K_{E}=\text{0}.\text{5},\;a_{E}=\text{1}\text{0},\;K_{L}=\text{2}.\text{5},\;v_{p}=\text{5}\text{5}\text{0},\;v_{n}=\text{8}\text{0},\;k_{pr}=\text{0}.\text{2},\;a_{L}=\text{2},\;a_{s}=\text{1}.\text{5},$$b_{s}=\text{0}.\text{0}\text{0}\text{1},a_{c}=\text{0}.\text{0}\text{5},\;b_{c}=\text{1},\;rV_{\text{0}}/V=\text{5}$*.*

In the numerical simulation, the time step was set to dt = 0.01 (min), and data on the migration and protrusions of 10 cells over a period of 600 minutes were collected as one set to create a distribution. At the start of the simulation, each cell was assigned three protrusions, with their generation directions determined randomly. The data from 60 minutes after the start to 660 minutes were used for analysis so that the period until cells began moving was excluded from the analysis. The model equations were solved numerically using the forward Euler method with a time step of dt. All simulations and analyses were performed using MATLAB R2024a (MathWorks, Inc.).

### Statistical analysis using mixed-effects models

To account for the hierarchical structure of the data, we analyzed each feature using mixed-effects models with either ECM or the value of $k_{pr}$ as a fixed effect and cell identity as a random effect (Figs 1 and 5). For continuous variables, including migration speed, turning angle, protrusion lifetime, maximum protrusion length, and mechanical polarity, we used linear mixed-effects models (LMM) of the form  Y~ ECM +(1 | CellID) or $Y\sim \;k_{pr}+\left(\text{1}\;|\text{ CellID}\right)$. For protrusion number, which represents count data, we used a generalized linear mixed-effects model (GLMM) with a Poisson distribution and a log link function, again with ECM or $k_{pr}$ condition as a fixed effect and cell identity as a random effect. The GLMM was fitted using the Laplace approximation. Statistical analyses were performed in MATLAB R2024a (MathWorks, Inc.).

## Supporting information

S1 Appendix Texts A-E, Figs A-M, and Tables A-I. (PDF)

S1 Movie Cell migration on fibronectin. GDC40 migration on a 2D plane on a fibronectin-coated matrix. The matrix concentration gradient was uniform.
(AVI)

S2 Movie Cell migration on laminin. GDC40 migration on a 2D plane on a laminin-coated matrix. The matrix concentration gradient was uniform.
(AVI)

S3 Movie Cell migration in simulation (F-ECM condition). Model simulation video assuming cell migration on fibronectin. Parameters estimated from images of GDC40 cell migration on fibronectin were used (F-ECM condition).
(AVI)

S4 Movie Cell migration in simulation (L-ECM condition). Model simulation video assuming cell migration on laminin. Parameters estimated from images of GDC40 cell migration on laminin were used (L-ECM condition).
(AVI)
