# Supplementary material for: Mechanical polarity links adhesion-regulated protrusions to directional stability in glioblastoma cell migration
Source: PLoS Comput Biol. 2026 Sep 24;22(9):e1014772. doi: 10.1371/journal.pcbi.1014772 (PMC13606977; doi:10.1371/journal.pcbi.1014772)
Supplement: Supplementary file 1 [file pcbi.1014772.s001.pdf]

S1 Appendix for
Mechanical polarity links adhesion-regulated protrusions to directional stability in glioblastoma cell migration

Haruna Tagawa[1&], Daisuke Kanematsu[2&], Asako Katsuma[2], Naoyuki Inagaki[1], Yonehiro Kanemura[2*], Yuichi Sakumura[1,3*]

1. Graduate School of Science and Technology, Nara Institute of Science and Technology, Ikoma, Japan
2. Department of Biomedical Research and Innovation, Institute for Clinical Research, NHO Osaka National Hospital, Osaka, Japan
3. Data Science Center, Nara Institute of Science and Technology, Ikoma, Japan

[&]H.T. and D.K. contributed equally to this work.
*kanemura.yonehiro.hk@mail.hosp.go.jp (Y.K), saku@bs.naist.jp (Y.S)

This PDF file includes:

Supporting Texts A-E
Figures A-M
Tables A-I
Legends for S1-S4 Movies
SI References

Other supporting materials for this manuscript include the following:

S1-S4 Movies

Supporting Information Text

Text A: Image processing procedure

1. Acquisition of cell body center coordinates

The image processing procedures performed before manual correction of cell body coordinates are shown in **Fig I**. First, each frame in the original time-lapse sequence was normalized, followed by intensity inversion and binarization. Connected components smaller than 100 pixels were removed as noise, and holes within the segmented regions were filled to allow identification of the cell body as a single connected component. In cases where multiple cells appeared in the same image, the target cell body was manually selected in the first frame. In subsequent frames, the region with the greatest overlap with the previously selected area was tracked as the same cell. Within the segmented region of the target cell, the largest inscribed circle [1] was defined as the core region of the cell body, and its center was used as the cell body center. However, due to occasional missegmentation or displacement of the cell body region, we manually verified and corrected the center coordinates after this series of processing steps.

2. Branch-like protrusion removal

In addition to protrusions extending from the cell body, branch-like protrusions can emerge from other protrusions. For simplicity, such “branch” structures were manually excluded from the analysis (**panel A of Fig J**).

3. Protrusion identification

To record the lifetime and the maximum length of each protrusion during its existence, it was necessary to identify individual protrusions over time. For this purpose, protrusions that appeared to be the same across consecutive image frames were assigned the same identification number (**panel B of Fig J**).

Initial identification was performed automatically. The procedure, illustrated in **panel C of Fig J**, involved comparing protrusion coordinates between consecutive frames—referred to as the previous image and the current image—to determine whether a given protrusion in the current image corresponded to one in the previous frame or represented a new one. The previous and current images were aligned based on the cell body center. For each candidate pair (a current protrusion and a protrusion in the previous image), two criteria were evaluated: (criterion 1) the distance between the coordinates near the base of the protrusion, and (criterion 2) the difference in the total number of coordinates, which approximates the protrusion length. Among the candidate protrusions in the previous image that met both criteria, the one with the smallest base distance (criterion 1) was considered to be the same protrusion and was assigned the same identifier. If no candidate in the previous frame satisfied both conditions, the current protrusion was treated as a new one and assigned a new identifier. This process was applied to each protrusion in each image. After automatic processing, manual inspection and corrections were performed as needed.

Text B: Additions and modifications to the neuronal polarity formation model for constructing the glioblastoma cell migration model

We constructed a cell migration model of GDC40 by adding to and modifying several elements of the neuronal polarity formation model [2]. The six major additions and modifications are as follows.

1. Non-probabilistic clutch molecule transport

Unlike neurons, probabilistic transport of clutch molecules to the tips of protrusions has not been observed in glioblastoma cells. Therefore, in glioblastoma cells, we introduced a deterministic molecular transport proportional to the concentration in the cell body, C_0. The dynamics of clutch molecule concentration at the protrusion tips C in the glioblastoma cell model are as follows:

$$V\frac{dC}{dt} = -\frac{AD}{L}(C - C_0) + rV_0C_0 ,$$

where constants A, D, V, and V_0 represent the cross-sectional area of the protrusion, the diffusion coefficient, the volume of the protrusion tip, and the volume of the cell body, respectively.

2. Constant total amount of cytoskeletal components and clutch molecules

In the neuronal polarity formation model, the expression levels of each molecule were changed over time based on the experimental data, as the model targeted neuronal polarity formation over several tens of hours. In this study, we focused on glioblastoma movement over 3–15 hours (**Table D**), so the total amount of

cytoskeletal components and clutch molecules in the cells was kept constant as E_m and E_C over time.

3. Introduction of the bound fraction function

In this model, to take into account the adhesive properties with respect to the extracellular matrix (ECM), the product of the protrusive force function $F_E(C)$ and the function $g(F_E)$ representing the bound fraction was used as the net protrusive force:

$$F_E(C)\, g(F_E) = \frac{a_E C^{h_E}}{{K_E}^{h_E} + C^{h_E}}\, g(F_E)\,.$$

Derivation of $g(F_E)$ is described later.

4. Movement of cell bodies due to pulling forces exerted by each protrusion

In the neuronal polarity formation model, the position of cell bodies was fixed. However, in this model, cell bodies were assumed to move due to pulling forces exerted by each protrusion (**Fig K**).

5. Model of protrusion formation and disappearance, and dynamics of cytoskeletal molecular components

In the neuronal polarity formation model, the number of protrusions does not change. We added factors for protrusion formation and disappearance to this model to vary the number of protrusions over time.

6. Modification of the dL/dt expression

Polymerization of cytoskeletal molecules is proportional to the amount of free cytoskeletal molecules M relative to the total cytoskeletal pool E_m. Unlike in the neuronal polarity formation model, the position of the cell body is not fixed in the glioblastoma cell migration model. Therefore, we expressed polymerization as the change in the protrusion tip position x_{tip} in absolute coordinates, rather than as the protrusion length L:

$$\frac{dx_{tip}}{dt} = v_{tip} = v_p \frac{M}{E_m} - v_n \exp\big(F_L - F_E \cdot g(F_E)\big)\ ,$$

where v_p and v_n are polymerization and depolymerization constants. F_L is a variable related to the tension of the protrusion.

Text C: Derivation of the bound fraction function g

Assume that the total number of adhesion molecules on the cell side is N_1, the number of adhesion sites on the extracellular matrix side is N_2, and N_b of these are bound. When $N_2 \gg N_b$, according to Bell et al. [3],

$$\frac{dN_b}{dt} = k_f(N_1 - N_b)N_2 - k_b N_b \exp\left(\frac{\gamma_s F}{K_B T N_b}\right).$$

Here, k_f and k_b are reaction rate constants (s^{-1}), K_B is the Boltzmann constant (m^2 kg s^{-2} K^{-1}), T is the absolute temperature (K), γ_s is a parameter determining the strength of the bond (μm) [4], and F is the force acting on the binding site (N).

When the effect of catch bond [5,6] is taken into account, it becomes

$$\frac{dN_b}{dt} = k_f(N_1 - N_b)N_2 - k_b N_b \exp\left(\frac{\gamma_s F}{K_B T N_b}\right) - k_{b,2} N_b \exp\left(\frac{-\gamma_c F}{K_B T N_b}\right).$$

Note that $\gamma_s > 0$ and $\gamma_c > 0$.

In a steady state $\left(\frac{dN_b}{dt} = 0\right)$,

$$0 = k_f(N_1 - N_b)N_2 - k_b N_b \exp\left(\frac{\gamma_s F}{K_B T N_b}\right) - k_{b,2} N_b \exp\left(\frac{-\gamma_c F}{K_B T N_b}\right),$$

and after reformulating the equation,

$$N_b = \frac{N_1}{\frac{k_b}{k_f N_2}\, exp\left(\frac{\gamma_s F}{K_B T N_b}\right) + \frac{k_{b,2}}{k_f N_2}\, \exp\left(\frac{-\gamma_c F}{K_B T N_b}\right) + 1}.$$

Here, if the bound fraction is g, $N_b = N_1\, g$, so

$$N_1 g = \frac{N_1}{\frac{k_b}{k_f N_2}\, \exp\left(\frac{\gamma_s F}{K_B T N_1 g}\right) + \frac{k_{b,2}}{k_f N_2}\, \exp\left(\frac{-\gamma_c F}{K_B T N_1 g}\right) + 1},$$

and

$$g = \frac{1}{\frac{k_b}{k_f N_2}\, \exp\left(\frac{\gamma_s F}{K_B T N_1 g}\right) + \frac{k_{b,2}}{k_f N_2}\, \exp\left(\frac{-\gamma_c F}{K_B T N_1 g}\right) + 1}.$$

If F is the traction force $f_C\, g$ of the model [2], F can be expressed as

$$F = f_C\, g = \frac{K_B T}{\delta} F_E\, g\,.$$

Here, δ is the length (μm) per unit of the skeletal components. Therefore,

$$g = \frac{1}{\frac{k_b}{k_f N_2} \exp\left(\frac{\gamma_s}{N_1\, \delta}\, F_E\right) + \frac{k_{b,2}}{k_f N_2} \exp\left(\frac{-\gamma_c}{N_1\, \delta}\, F_E\right) + 1}$$

$$= \frac{1}{b_{\mathrm{s}} \exp(a_{\mathrm{s}}\, F_E) + b_{\mathrm{c}} \exp(-a_{\mathrm{c}}\, F_E) + 1},$$

where

$$a_{\mathrm{s}} = \frac{\gamma_s}{N_1 \delta}, \qquad a_{\mathrm{c}} = \frac{\gamma_c}{N_1 \delta}, \qquad b_{\mathrm{s}} = \frac{k_b}{k_f N_2}, \qquad b_{\mathrm{c}} = \frac{k_{b,2}}{k_f N_2}$$

are used. For simplicity, the number of adhesion molecules N_1 on the cell side is assumed to be constant.

Text D: Calculation method for JS divergence

JS divergence (Jensen-Shannon divergence) is a metric that represents the difference between two distributions $p(x)$ and $q(x)$. When $p(x)$ and $q(x)$ are discrete distributions, it can be calculated using the following formula:

$$JS(p||q) = \frac{1}{2} KL(p\ ||\ R) + \frac{1}{2} KL(q\ ||\ R)\,, \qquad (1)$$

where

$$R = \frac{p+q}{2}\,, \qquad (2)$$

$$KL(p||R) = \sum_x p(x) \log\left(\frac{p(x)}{R(x)}\right). \qquad (3)$$

In this study, $p(x)$ represents the distribution obtained from simulation results, and $q(x)$ represents the distribution obtained from quantitative results of images. The JS divergence was used as an indicator of how well the simulation results reproduce the actual data. Note that even for distributions with continuous values, such as migration speed and turning angle, they were treated as discrete values by dividing them into intervals of dx. The bin width dx was determined for each feature by applying the Freedman–Diaconis rule [7] to the distribution obtained from the images. The procedure for calculating the JS divergence between the distributions of the quantitative results from the images and the simulation results is as follows.

First, we normalized the distribution quantified from the simulation results and images by setting the bin

width to dx and approximating the distribution area to 1. We set the values of the bar heights at x in each histogram as $p(x)$ and $q(x)$. We calculated the JS divergence between $p(x)$ and $q(x)$ using the above formula, targeting the range from the smaller lower limit to the larger upper limit of the two distributions [8]. When $p(x) = 0$ or $q(x) = 0$, we replaced those values with 0.00001 (since the argument of the logarithm must be positive).

For the distribution-based fitting, histograms from experimental and simulated data were normalized before the JS divergence was computed, so the metric did not directly scale with the absolute number of samples. To examine the effect of cell number, we performed an additional subsampling analysis in which five cells were randomly selected from the ten simulated cells (100 times). In each of 100 iterations, five of the ten simulated cells were randomly selected without replacement from each ECM condition, and the corresponding feature distributions were recalculated. **Fig G** shows one representative iteration, whereas **Table I** summarizes the results across all 100 iterations. Statistically significant ECM-dependent differences in all five features were observed in 97 of the 100 iterations (**Table I**). The resulting distributions were similar to those obtained from all ten simulated cells (**panels B and C of Fig G**), and the ECM-dependent differences in motility-related features were also mostly preserved (**panel A of Fig G**). Thus, the subsampling analysis suggests that the mismatch in cell number did not qualitatively alter the main conclusions. Nevertheless, we note that finite-sample variability in the estimated distributions may still differ between experimental and simulated datasets when the sample sizes are not matched exactly.

Text E: Robustness of model conclusions across independently fitted parameter sets

To examine whether the main conclusions depended on a particular fitted parameter set, we performed 16 independent fitting runs starting from the same initial parameter values (**Fig B**). Owing to the stochastic nature of the fitting procedure, these runs converged to distinct parameter combinations, with several fitted values differing by more than 10% from their across-run means.

Despite this variability in the fitted parameters, the experimentally observed ECM-dependent phenotypic pattern was consistently reproduced across the independently obtained solutions. Specifically, among the four features that showed significant differences between the fibronectin and laminin conditions in the experimental data—turning angle, migration speed, protrusion number, and maximum protrusion length—at least three were reproduced in all 16 fitted solutions, and all four were reproduced in 7 of the 16 solutions (**Table H**).

In addition, the overall agreement between the model-predicted and experimental distributions was assessed using the summed Jensen–Shannon divergence across the five measured features. For the fibronectin-fitted model, the matched model–experiment comparison showed a lower summed JS divergence than the corresponding mismatched comparison in all 16 solutions. For the laminin-fitted model, this relationship was observed in 10 of the 16 solutions.

We also examined correlations among the fitted parameters across the 16 solutions and found that several parameters covaried, suggesting compensatory relationships among them (**Fig C**). Because the fitted parameters were not independent, perturbing one parameter at a time while holding all others fixed can generate parameter combinations that were not represented among the solutions supported by the fitting procedure. We therefore used the ensemble of independently fitted parameter sets to assess robustness across fitted parameter combinations while retaining these compensatory relationships.

These results indicate that, although the individual fitted parameter values are not unique, the overall ECM-dependent phenotypic pattern was relatively robust across distinct fitted parameter combinations. However, complete reproduction of all experimentally observed differences was not achieved in every solution, and the distribution-level agreement was less consistent for the laminin-fitted model.

Supporting figures

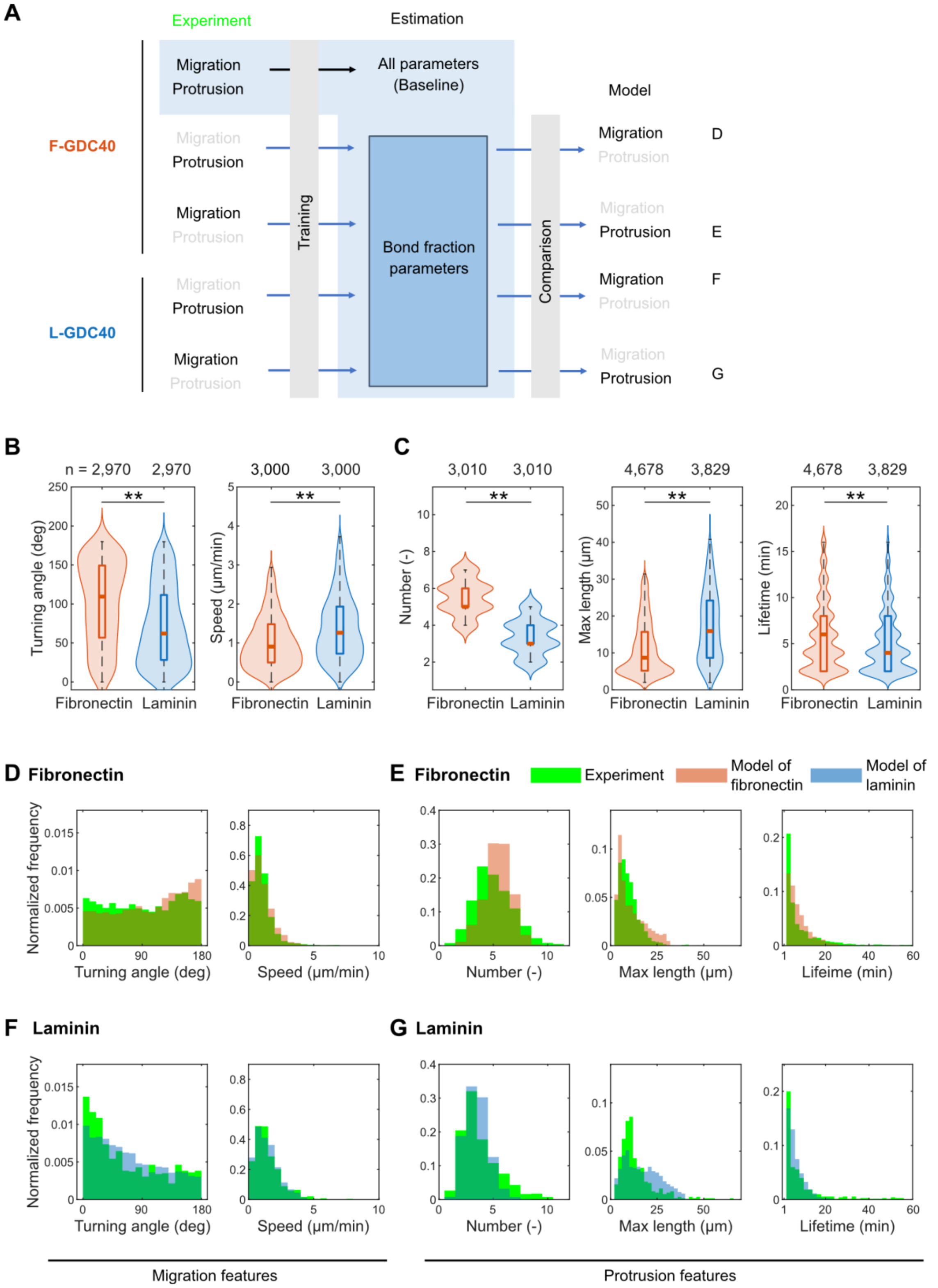

Fig A. Reproduction of migration features from protrusion feature data, and vice versa. (A) Using the F-ECM condition as the baseline, bound fraction parameters a_s, b_s, a_c, and b_c were estimated from the distribution of protrusion features (number, length, lifetime) for F-GDC40 or L-GDC40. The simulation results with the parameters are shown in (B), (D) and (F). Similarly, bound fraction parameters

were estimated from the distributions of migration features (turning angle and speed) for F-GDC40 or L-GDC40. The simulation results with the parameters are shown in (C), (E) and (G). For comparison, the distributions obtained from actual cell images (green; same as in **Fig 2C and D**) are also shown. Statistical differences were assessed using mixed-effects models with ECM condition as a fixed effect and cell identity as a random effect (**p < 0.01).

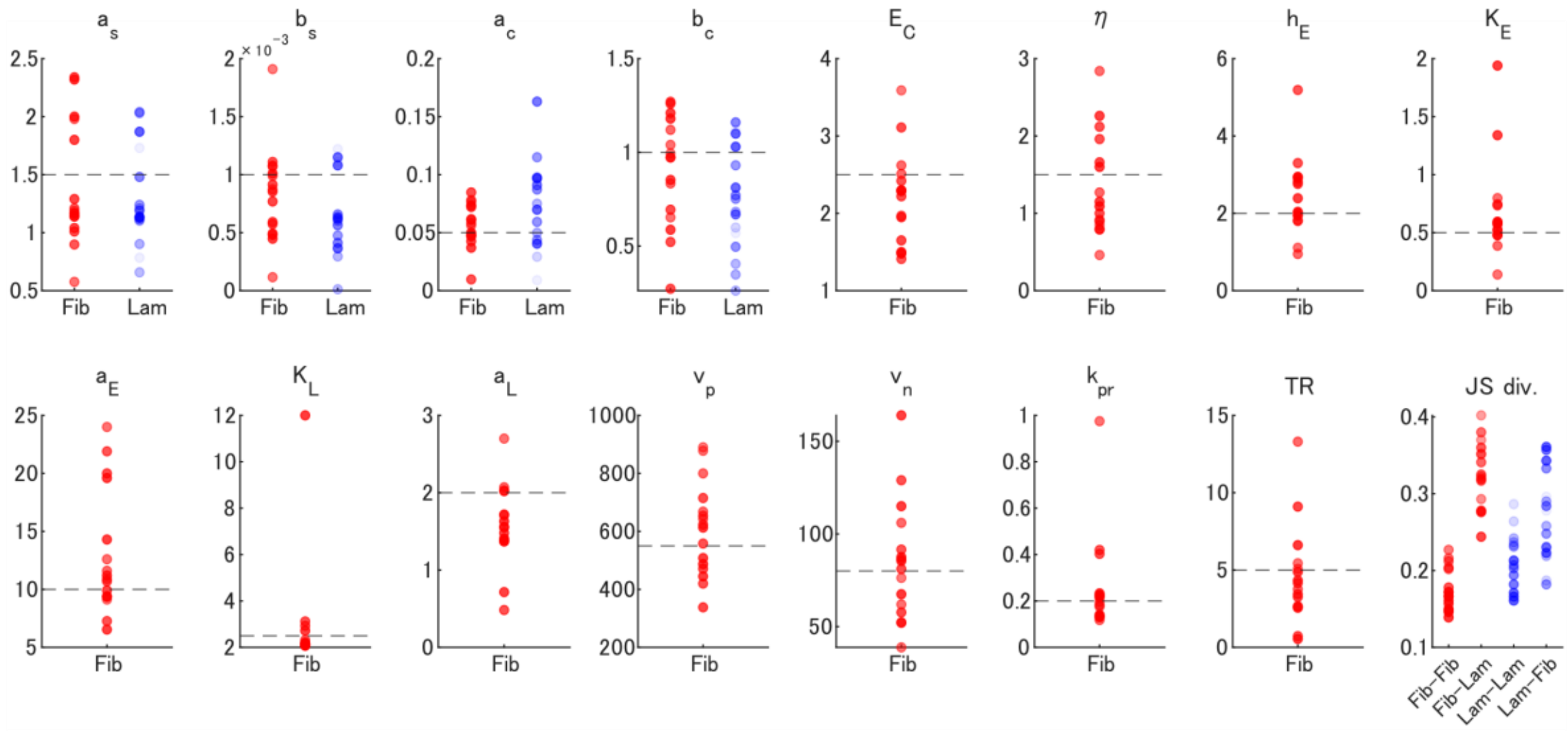

Fig B. Parameter estimation results from 16 independent fitting runs and corresponding JS divergences.

The dotted horizontal lines indicate the initial parameter values used for parameter estimation. “Fib” denotes the results obtained under the fibronectin condition, and “Lam” denotes the results obtained under the laminin condition. For the JS divergence plot, the label on the left indicates the ECM condition of the simulated distribution, whereas the label on the right indicates the ECM condition of the experimental distribution used for comparison. For example, “Fib-Lam” denotes the JS divergence between the simulated distributions under the fibronectin condition and the experimental distributions under the laminin condition. Darker points indicate parameter sets with lower JS divergence values for the corresponding matched comparison (“Fib-Fib” or “Lam-Lam”). “TR” represents the parameter $r\,V_0/V$. For details of the parameter estimation procedure, see the Methods section of the main text.

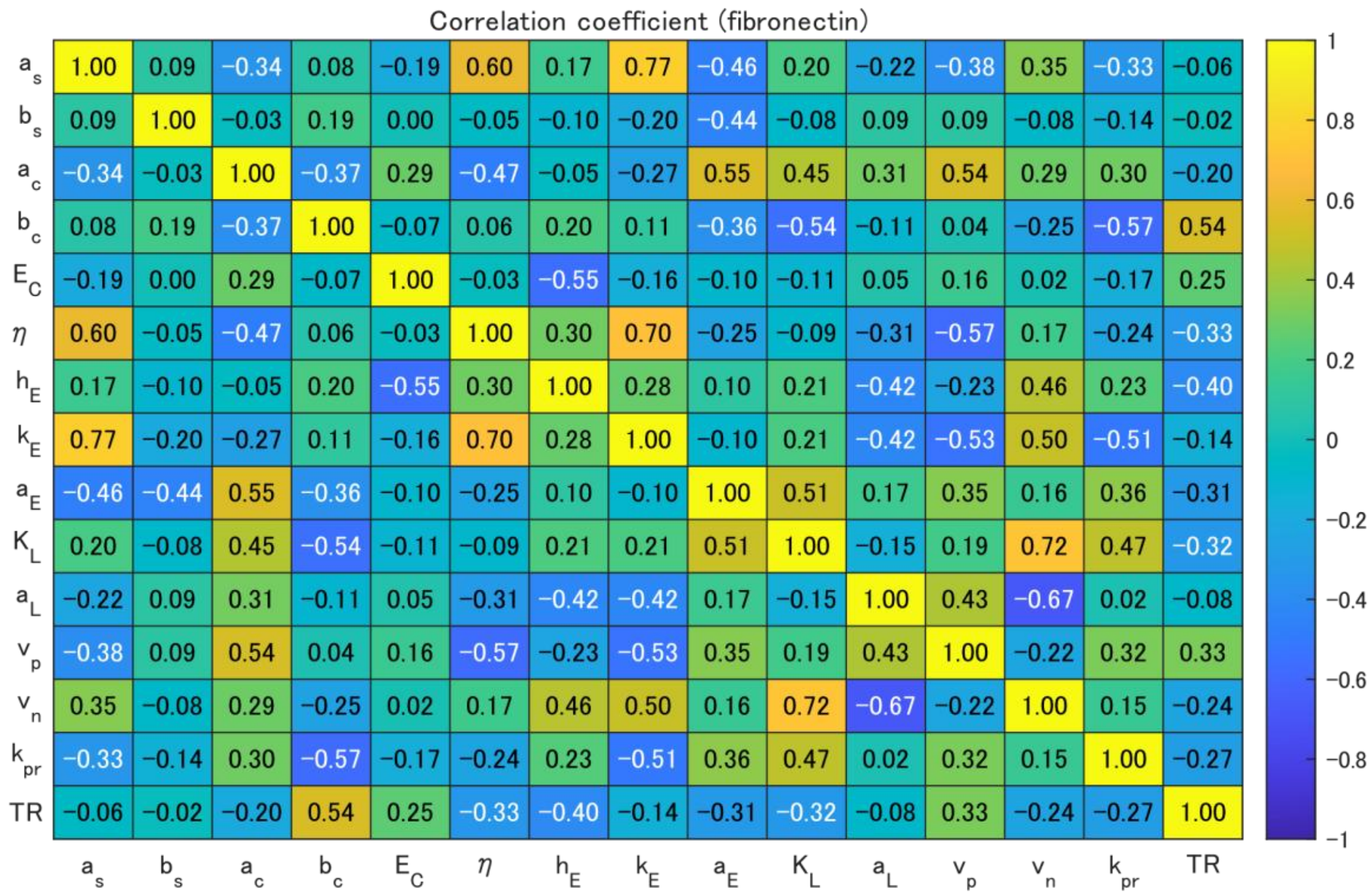

Fig C. Correlations among parameters from 16 independent fitting runs under the fibronectin conditions.

This heat map was generated from the fibronectin parameter estimates shown in **Fig B**. Colors and values indicate Spearman's rank correlation coefficients between pairs of fitted parameters. "TR" represents the parameter $r\,V_0/V$.

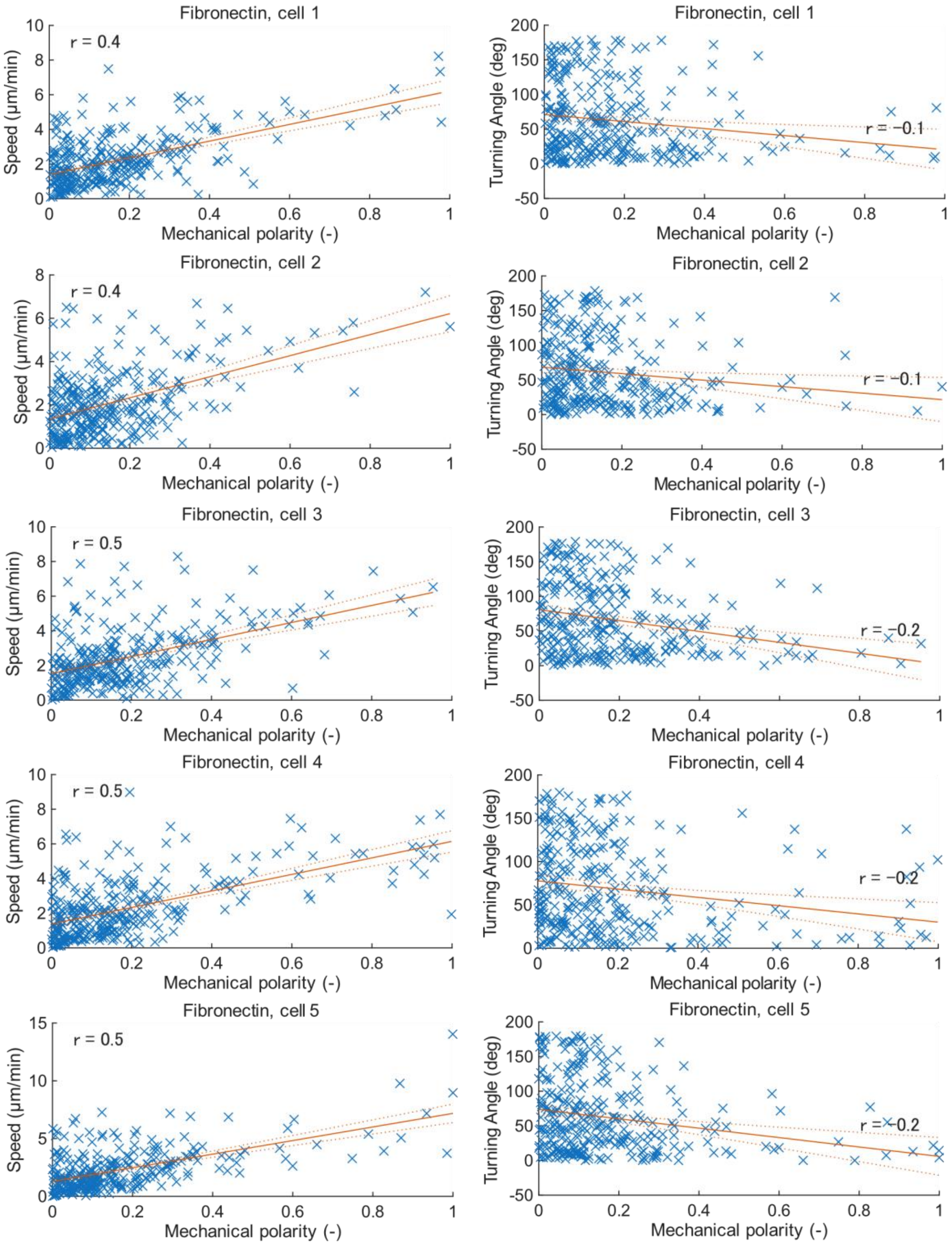

Fig D. Relationship between mechanical polarity and dynamic migration features (fibronectin). The relationship between mechanical polarity and dynamic migration features was quantitatively evaluated for each simulated cell on fibronectin. Mechanical polarity and the corresponding speed and turning-angle measures were calculated every 2 minutes over 600 minutes of simulation, and their correlations were assessed using Spearman's correlation coefficient (r). Solid lines indicate linear regression lines, and dotted lines indicate the 95% confidence intervals. Results from 5 of the 10 simulated cells under the fibronectin condition are shown.

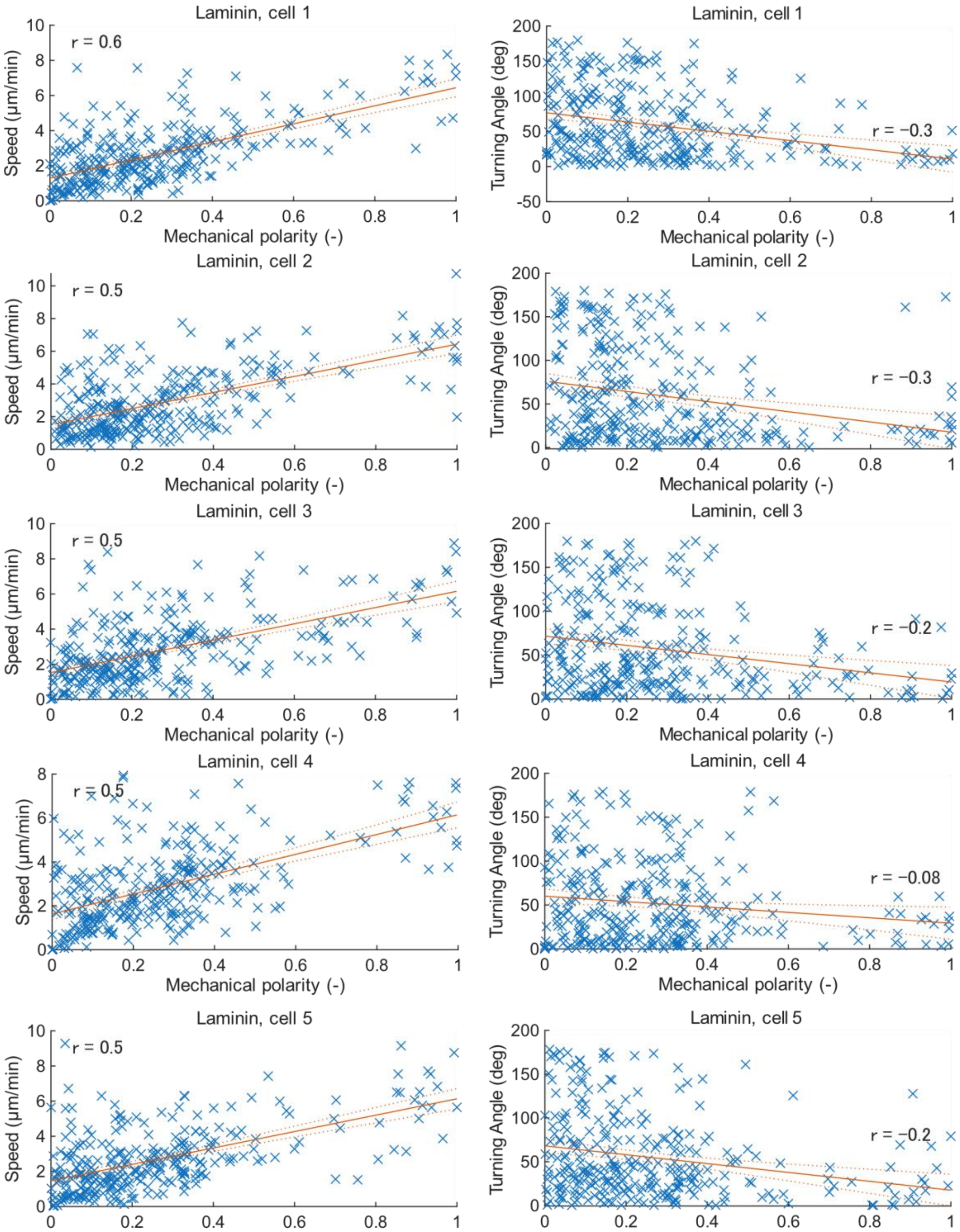

Fig E. Relationship between mechanical polarity and dynamic migration features (laminin). Same as **Fig D**, but for the laminin condition instead of the fibronectin condition.

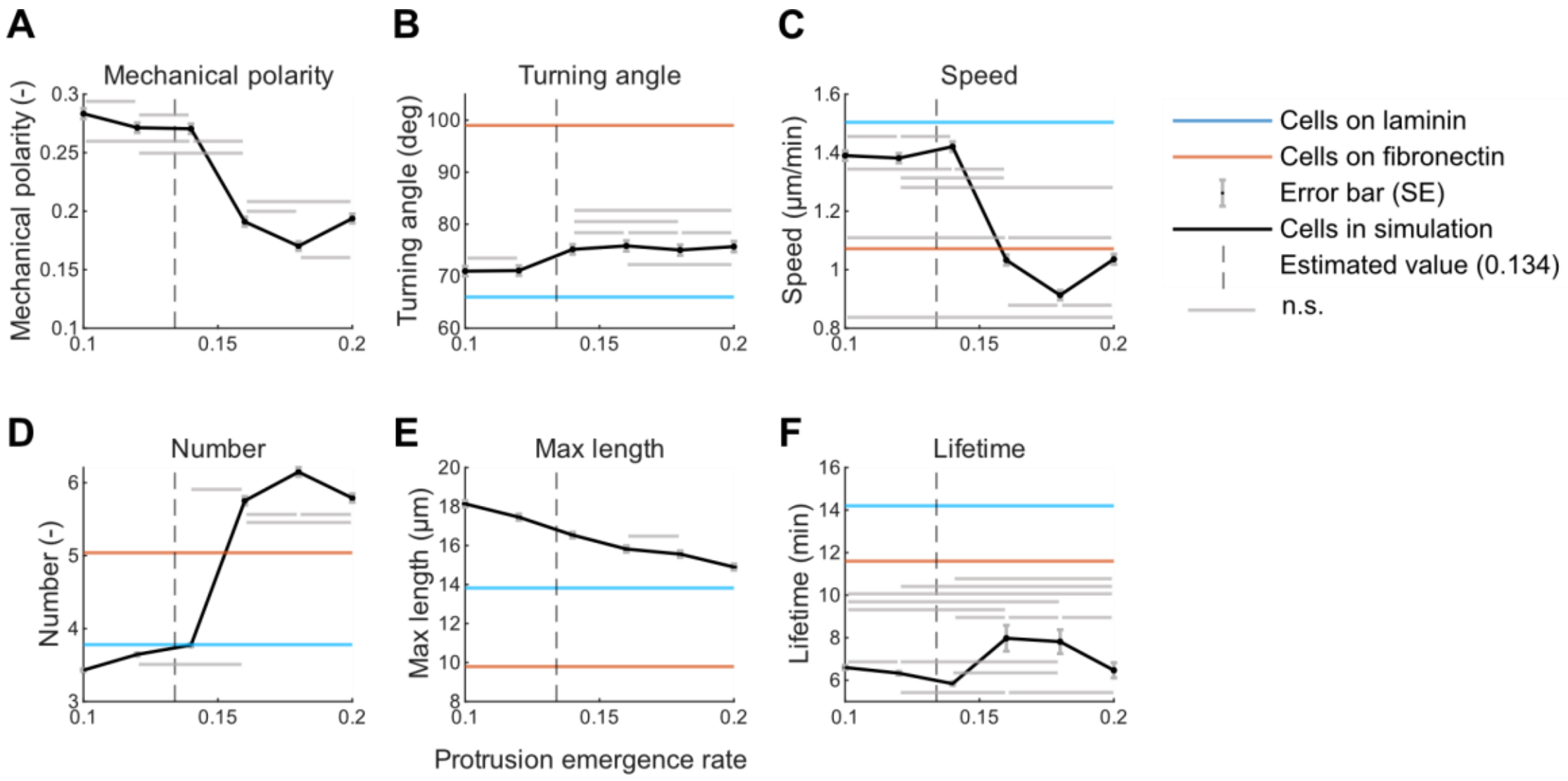

Fig F. Sensitivity of migration features to protrusion emergence rate coefficient k_{pr} .
Simulations were performed for k_{pr} values from 0.1 to 0.2 (increment 0.02), with 10 cells simulated per condition for 600 minutes. Data were recorded every 2 minutes, and each panel shows mean ± standard error. (A–F) Mechanical polarity, turning angle, migration speed, protrusion number, maximum protrusion length, and lifetime, respectively. Orange and blue lines represent the experimental means of F-GDC40 and L-GDC40, respectively. The vertical dashed line indicates the fitted value of k_{pr} for F-GDC40 ($k_{pr} = 0.134$). Statistical differences were assessed using mixed-effects models, with protrusion emergence rate as a fixed effect and cell identity as a random effect, and p values for pairwise comparisons were adjusted for multiple testing using the Holm method. The gray horizontal lines indicate combinations that did not show significant differences ($p > 0.05$).

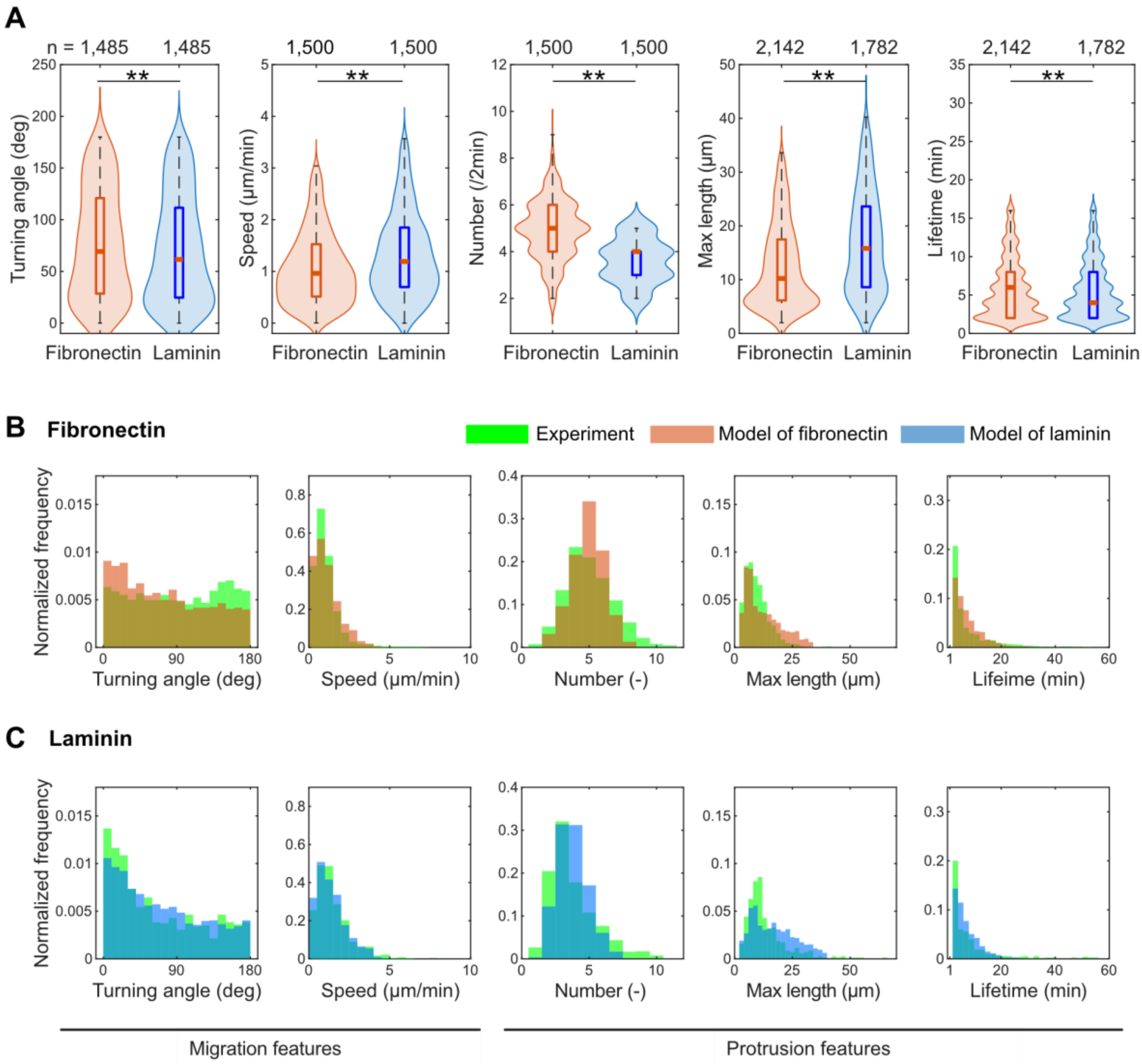

Fig G. Motility-related simulation results based on five randomly selected cells per ECM condition. Five of the 10 simulated cells were randomly selected without replacement from each ECM condition. The main text presents simulation results based on all 10 cells per condition (**Fig 1G, and Fig 2C and D**). Results from one of the 100 random-subsampling iterations are shown here, whereas the results from all 100 iterations are summarized in **Table I**. The histograms of the experimental data (green) are identical to those shown in **Fig 2C and D**. Statistical significance was assessed using mixed-effects models with ECM condition as a fixed effect and cell identity as a random effect (**$p < 0.01$).

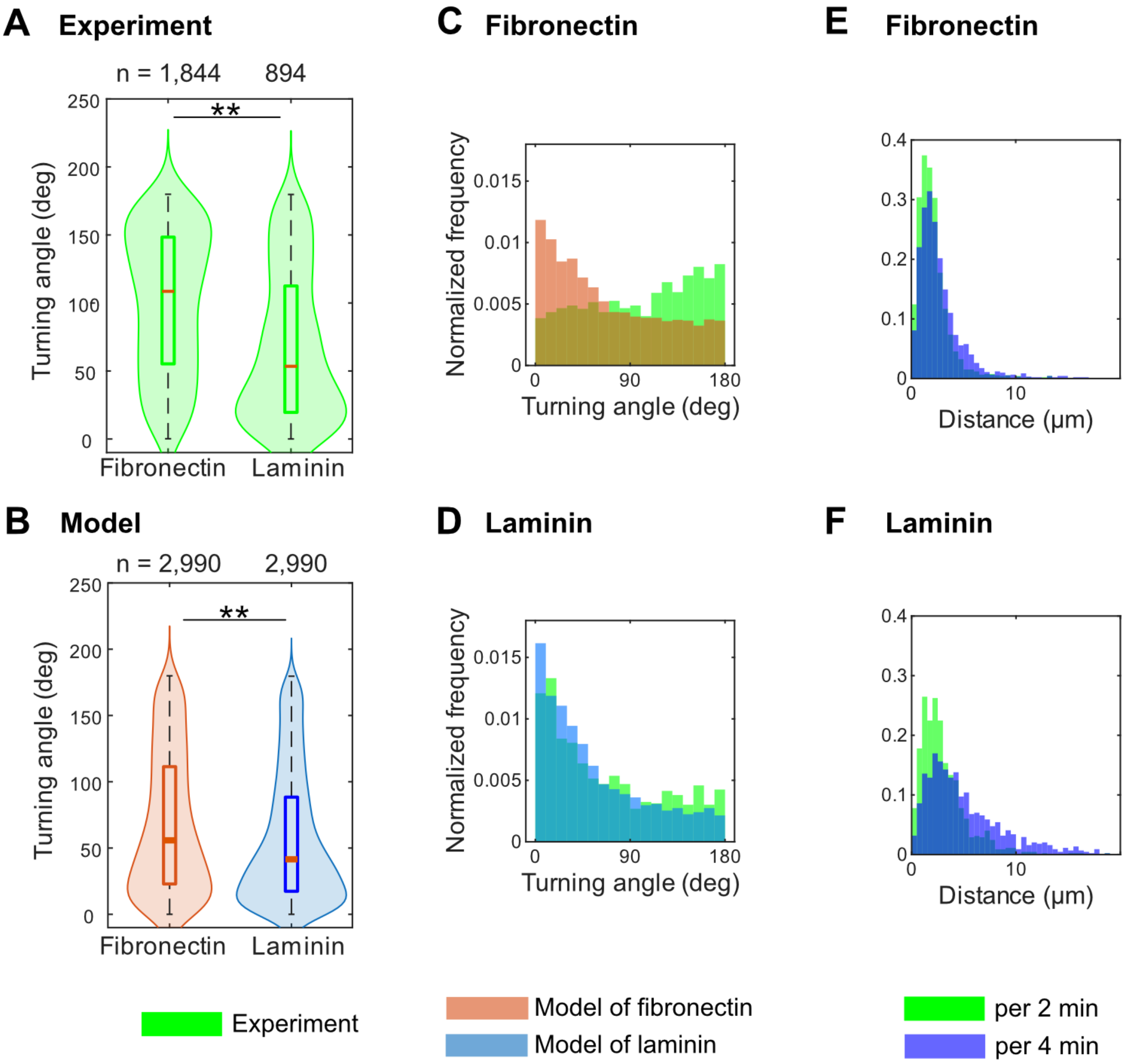

Fig H. Turning angles at 2-minute intervals and distances at 2-minute and 4-minute intervals. (A, B) Violin plots of turning angles at 2-minute intervals: (A) shows results obtained from actual cell images, and (B) shows results from model simulations. The orange violin represents simulation results for cells on fibronectin (F-ECM condition; see main text), while the blue violin represents results for cells on laminin (L-ECM condition; see main text). Statistical significance was assessed using linear mixed-effects models (LMM) with ECM condition as a fixed effect and cell identity as a random effect (**$p < 0.01$). (C, D) Comparison of histograms of (A, B). Results for cells on fibronectin (C) and on laminin (D) are shown. (E, F) Histogram showing cell body displacement measured at 2-minute (green) or 4-minute (dark blue) intervals. Both results were obtained from actual cell images.

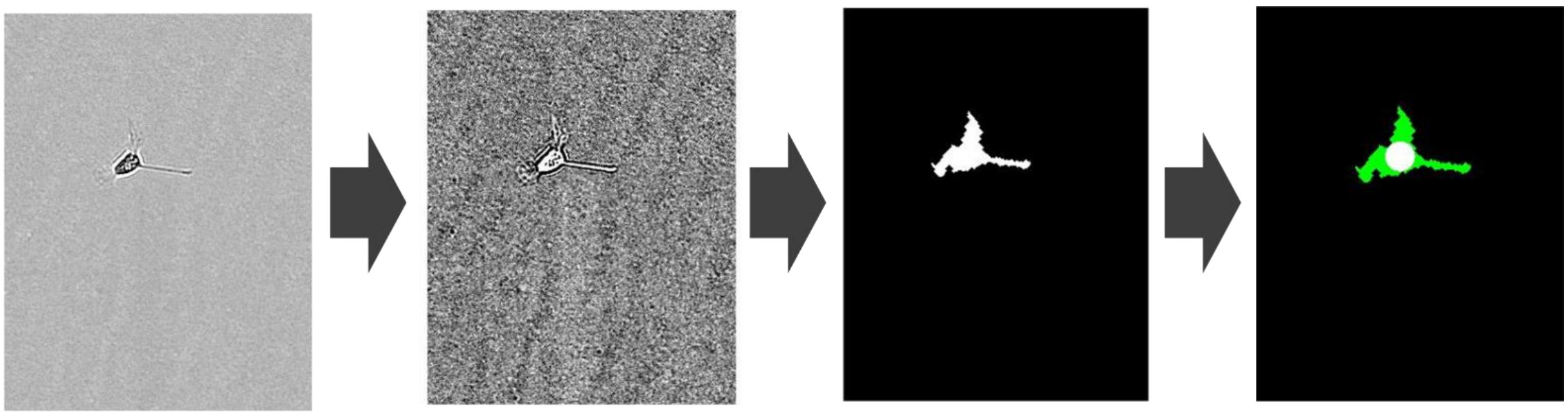

Fig I. Method for automated extraction of cell body center coordinates.
The original images were first normalized, followed by intensity inversion and binarization. Noise was removed, and holes were filled to enable identification of the target cell body region as a connected component. Since multiple cells may appear in a single image, the target cell was manually selected in the initial frame. In subsequent frames, the region with the greatest overlap with the previously selected region was tracked as the same cell. The center of the largest inscribed circle within the identified region was defined as the center of the cell body.

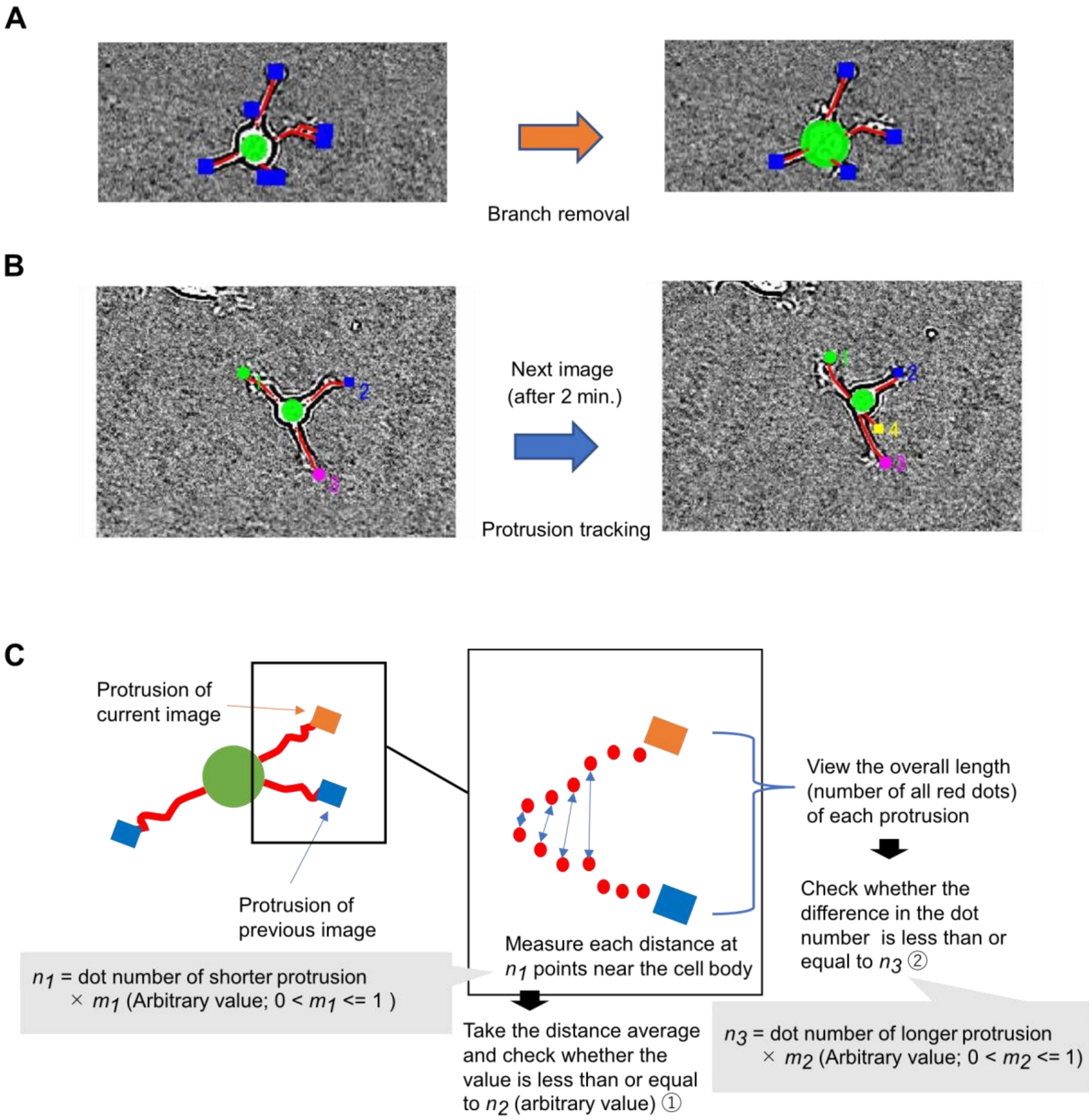

Fig J. Processing of cellular protrusions.
(A) Removal of branch-like structures. Protrusions that appeared to branch off from other protrusions, rather than directly extending from the cell body, were excluded from the analysis as branch-like structures. (B) Protrusion identification. The same identification number was assigned to protrusions that were judged to be the same across consecutive frames to identify individual protrusions across image frames. (C) Automatic method for protrusion identification. This panel illustrates how each protrusion in the current image (orange) was compared to protrusions in the previous image to determine whether it was the same or newly formed. The previous and current images were aligned based on the center of the cell body. For each pair consisting of a protrusion from the current image and one from the previous image, two conditions were evaluated: [1] whether the distances between the base coordinates near the cell body (specifically, the closest n_1 coordinates) were within a defined threshold n_2, [2] whether the difference in the total number of coordinates, which corresponds to protrusion length, was within a defined threshold n_3. Among the candidate protrusions in the previous image that met both conditions, the one with the smallest base distance was considered to be the same protrusion and was assigned the same identification number. If no candidate met both conditions, the protrusion was treated as a new one and assigned a new identifier. The parameter values used in this analysis were $n_2 = 25, m_1 = 0.8$, and $m_2 = 0.5$.

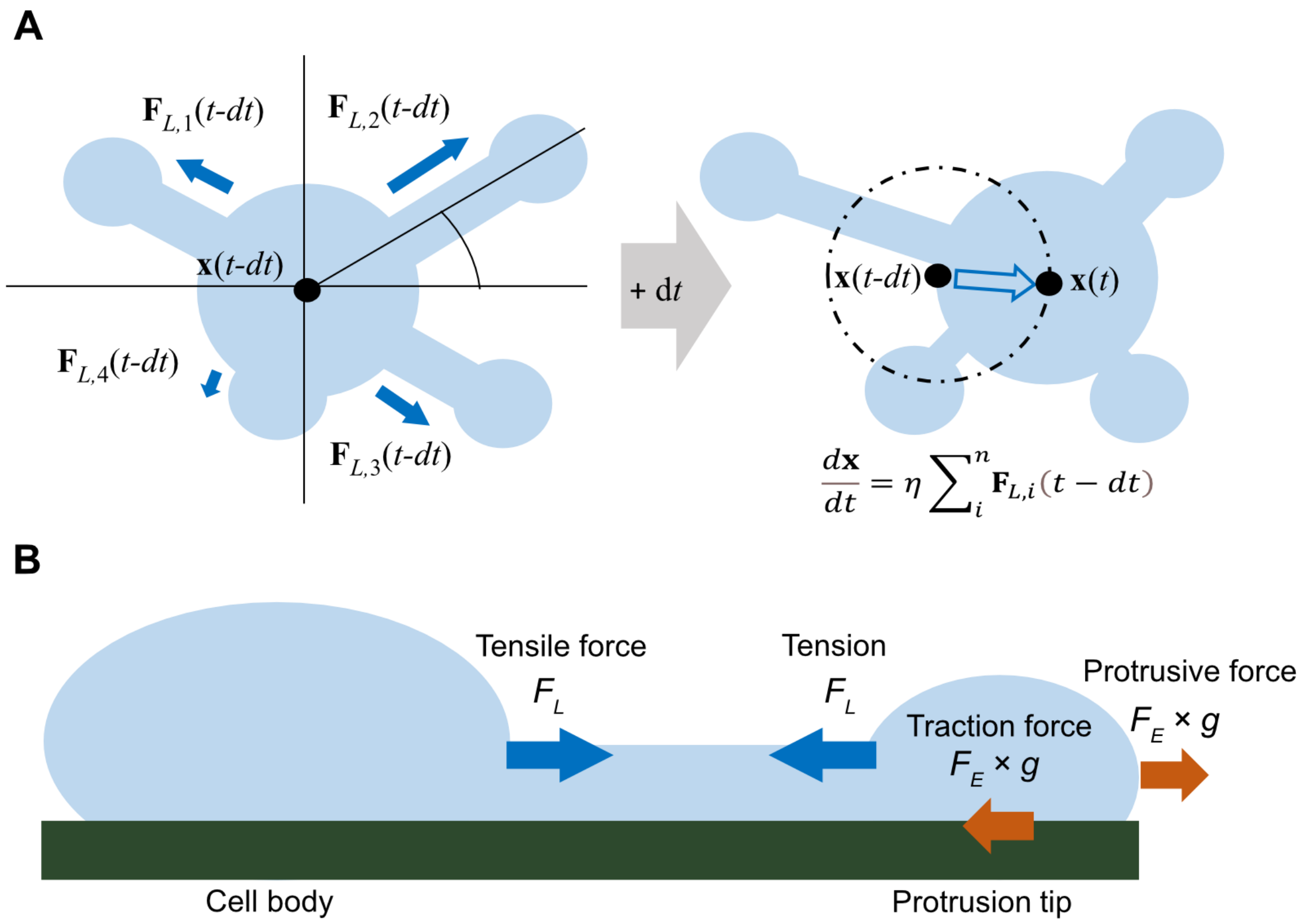

Fig K. Displacement of the cell body position x due to protrusion-generated pulling forces. (A) The cell body migrates in response to the tensile forces $\mathbf{F}_L$ generated by individual protrusions. The parameter η is the motility coefficient associated with cell body movement. (B) While protrusion tip extension is driven by the protrusive force $F_E\ g$, which results from the traction force to substrate resistance via actin retrograde flow, the migration of the cell body is governed by the tensile force F_L acting along the protrusion shaft. The tension applied during protrusion elongation promotes retraction.

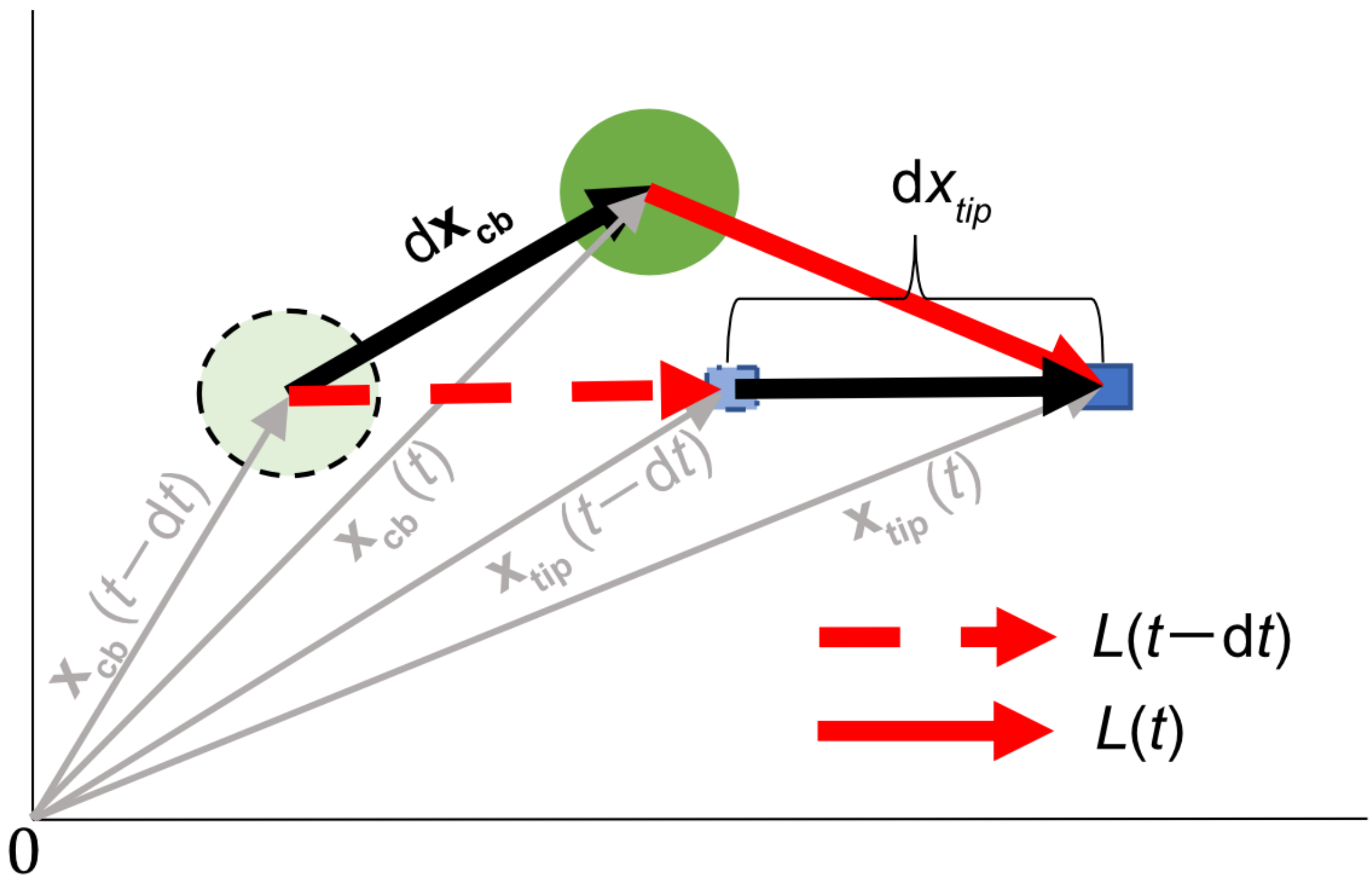

Fig L. Cell body movement and protrusion elongation.

At time point $t - dt$, $\mathbf{x}_{\mathrm{cb}}(t - dt)$ is the vector from the origin to the center of the cell body, and $\mathbf{x}_{\mathrm{tip}}(t - dt)$ is the vector from the origin to the tip of the protrusion. After a time interval $\mathrm{d}t$, if the protrusion elongates by $\mathrm{d}x_{tip}$, the vector from the origin to the tip becomes $\mathbf{x}_{\mathrm{tip}}(t)$. If the cell body does not move during this interval, the new protrusion length becomes the original length $L(t - dt)$ (indicated by the red dashed line) plus $\mathrm{d}x_{tip}$. However, if the cell body moves by $\mathrm{d}\mathbf{x}_{\mathrm{cb}}$, resulting in a new vector $\mathbf{x}_{\mathrm{cb}}(t)$, then the protrusion length is updated accordingly. The final length $L(t)$ is given by the magnitude of the updated vector from the new cell body center to the tip, as illustrated by the red solid line in the figure.

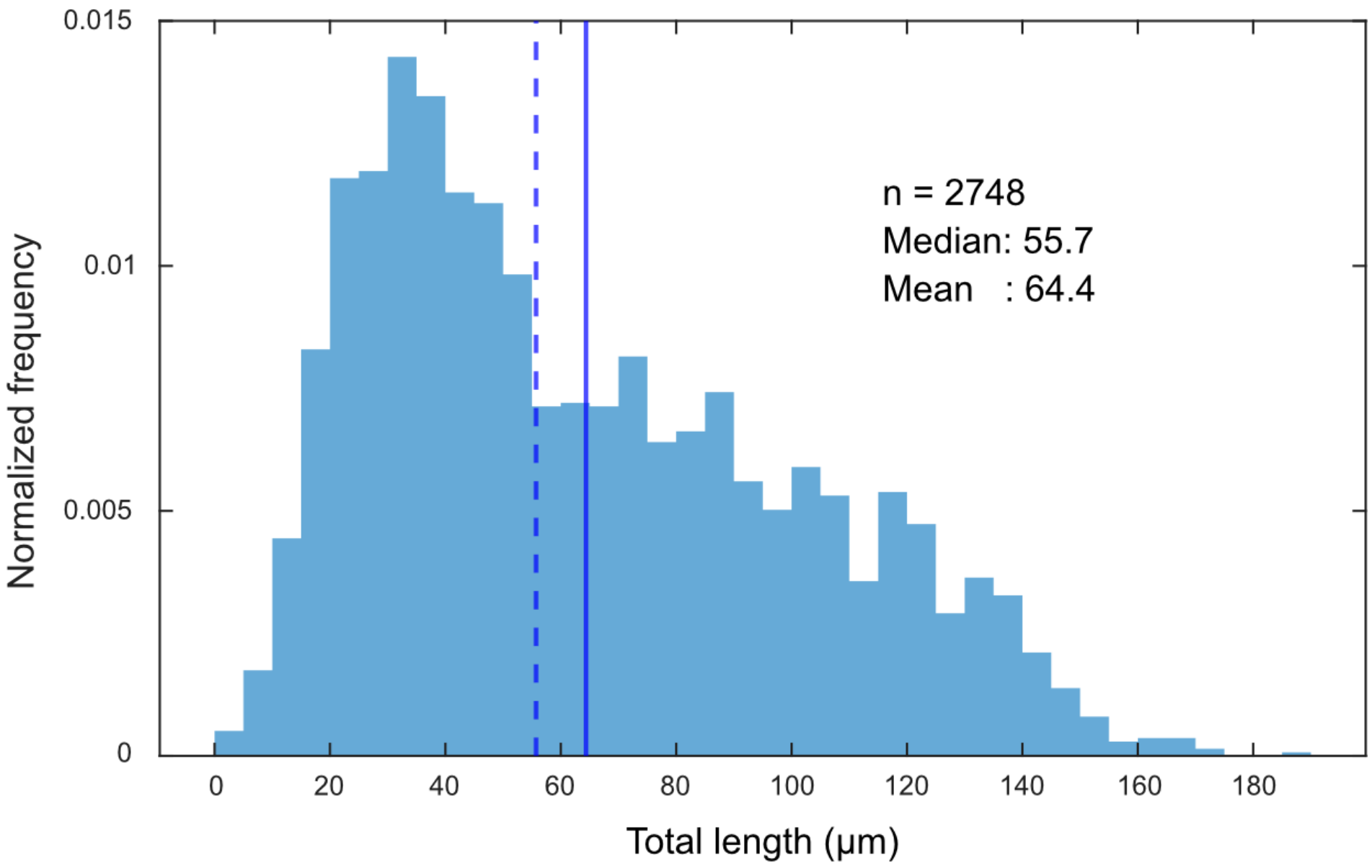

Fig M. Total protrusion length per cell measured every 2 minutes in GDC40 cells. Protrusion length was defined as the straight-line distance from the base to the tip of each protrusion. Results from 10 cells were compiled for both fibronectin and laminin ECM conditions. n indicates the number of samples. The median (blue dashed line) and mean (blue solid line) are shown. In our cell migration model, the parameter E_m, representing the total length of cytoskeletal components within a cell, was set to the average of these total protrusion lengths (64.4 μm).

Supporting tables

Table A. Jensen–Shannon (JS) divergences comparing model feature distributions with experimental data under each ECM condition.

Values are shown for the JS divergence between the F-ECM model distribution (orange in **Fig 2C**) and the actual F-GDC40 distribution (green in **Fig 2C**) or the actual L-GDC40 distribution (green in **Fig 2D**), and the JS divergence between the L-ECM model distribution (blue in **Fig 2D**) and the actual F-GDC40 distribution (green in **Fig 2C**) or the actual L-GDC40 distribution (green in **Fig 2D**).

JS divergence of model vs experiment	Turning angle	Speed	Number	Max length	Lifetime
Fibronectin vs fibronectin	0.009	0.018	0.031	0.046	0.039
Fibronectin vs laminin	0.012	0.026	0.134	0.049	0.053
Laminin vs fibronectin	0.020	0.042	0.098	0.120	0.046
Laminin vs laminin	0.006	0.010	0.040	0.055	0.061

Table B. JS divergences comparing model feature distributions with experimental data when adhesion parameters were estimated using only migration or protrusion features.

Values are shown for the JS divergence between the F-ECM model distribution (orange in **panels D and E of Fig A**) and the actual F-GDC40 distribution (green in **Fig 2C**) or the actual L-GDC40 distribution (green in **Fig 2D**), and the JS divergence between the L-ECM model distribution (blue in **panels F and G of Fig A**) and the actual F-GDC40 distribution (green in **Fig 2C**) or the actual L-GDC40 distribution (green in **Fig 2D**).

JS divergence of model vs experiment	Turning angle	Speed	Number	Max length	Lifetime
Fibronectin vs Fibronectin	0.010	0.016	0.049	0.034	0.044
Fibronectin vs Laminin	0.010	0.030	0.193	0.068	0.058
Laminin vs Fibronectin	0.020	0.047	0.132	0.123	0.052
Laminin vs Laminin	0.009	0.009	0.036	0.058	0.063

Table C. JS divergences comparing model feature distributions when the protrusion emergence rate is increased.

Values are shown for the JS divergence between the simulated distribution at $k_{pr} = 0.2$ (magenta in **Fig 5**) and the distribution for F-GDC40 (green in **Fig 2C**) or the distribution for L-GDC40 (green in **Fig 2D**).

JS divergence of model ($k_{pr} = 0.2$) vs experiment	Turning angle	Speed	Number	Max length	Lifetime
vs fibronectin	0.009	0.093	0.101	0.085	0.052
vs laminin	0.011	0.051	0.115	0.060	0.061

Table D. Cell labels and corresponding number of image frames.

"Fib cell x" indicates a cell on fibronectin-coated substrate, and "Lam cell x" indicates a cell on laminin-coated substrate.

Cell label	Number of images	Cell label	Number of images
fib cell 1	320	lam cell 6	235
fib cell 2	452	lam cell 7	180
fib cell 3	452	lam cell 8	130
fib cell 4	280	lam cell 9	100
fib cell 5	350	lam cell 10	259

Table E. Summary of parameters used in the glioblastoma migration model. The parameter E_m was estimated from the average total protrusion length per time point, based on 2-minute-interval time-lapse images (a total of 2,748 frames) from 10 glioblastoma cells (see **Fig M**). Parameters $r\,V_0/V$, $v_p, v_n, E_C, \eta, k_{pr}, a_E, h_E, K_E, a_L, K_L, a_s, b_s, a_c$ and b_c were estimated from the distributions of quantitative features obtained from GDC40 cells migrating on fibronectin (F-ECM condition; see main text). The parameters a_s, b_s, a_c and b_c were also estimated from data on laminin-coated substrates, while the values of the other parameters were fixed to match those of the F-ECM (L-ECM condition; see main text). The parameter $A\,D\,/\,V$ was taken from the previous study [2]. Fixed or externally determined parameter values are indicated as follows: † taken from Ref. [2]; ‡ estimated from the experimental measurements shown in **Fig M**; § empirically adjusted; ¶ fixed at the value fitted under the F-ECM condition.

Parameter	Unit	Value		Meaning
		F-ECM	L-ECM	
$A\,D\,/\,V$	µm/min	8.26†	8.26†	Effective diffusion coefficient per unit length. A, D, and V represent the cross-sectional area of the protrusion, the diffusion coefficient of the clutch molecule, and the volume of the protrusion tip, respectively.
$r\,V_0/V$	/min	4.88	4.88¶	The ratio (r) of clutch molecules transported per unit time from the cell body, and the coefficient for concentration conversion based on the volume ratio between the cell body and the protrusion tip ($V\,/V_0 = 1/10$).
v_p	µm/min	508	508¶	Polymerization rate constant of cytoskeletal components
v_n	µm/min	52.1	52.1¶	Depolymerization rate constant of cytoskeletal components
E_m	µm	64.4‡	64.4‡	Total amount of cytoskeletal components in whole cell
E_C	Relative conc.	1.48	1.48¶	Total amount of clutch molecules in whole cell
η	µm/min	1.15	1.15¶	Cell motility coefficient
k_{pr}	-	0.134	0.134¶	Coefficient at protrusion emergence rate k_p
a_E	-	6.54	6.54¶	Maximum value of F_E
h_E	-	2.04	2.04¶	Hill coefficient
K_E	Relative conc.	0.595	0.595¶	Half-value constant of F_E
a_L	-	1.71	1.71¶	Maximum value of F_L
K_L	µm	2.07	2.07¶	Half-value constant of F_L
L_0	µm	2§	2§	Initial protrusion length and removal threshold
a_s	-	2.00	1.87	Parameter of function g
b_s	-	8.54×10^{-4}	6.19×10^{-4}	Parameter of function g
a_c	-	3.70×10^{-2}	4.01×10^{-2}	Parameter of function g
b_c	-	1.21	1.03	Parameter of function g

Table F. Variables in the glioblastoma migration model

Variable	Unit	Meaning
C_i	Relative conc.	Concentration of clutch molecules at the tip of protrusion i
C_0	Relative conc.	Concentration of clutch molecules in the cell body
M	μm	Total amount of free cytoskeletal components (total length)
L_i	μm	Length of protrusion i
t	min	Time in simulation

Table G. Expressions in the glioblastoma migration model

Formula	Meaning
$v_{tip} = v_p \frac{M}{E_m} - v_n \exp\left(F_L - F_E \cdot g(F_E)\right)$	Speed of protrusion elongation at the protrusion tip
$F_E(C) = \frac{a_E C^{h_E}}{{K_E}^{h_E} + C^{h_E}}$	Effect of protrusive force on the concentration of clutch molecules at the protrusion tip
$F_L(L) = \frac{a_L \ln(L/L_0)}{\ln(K_L/L_0) + \ln(L/L_0)}$	Effect of stress on protrusion retraction depending on protrusion length
$g(F_E) = \frac{1}{b_s e^{a_s F_E} + b_c e^{-a_c F_E} + 1}$	Bound fraction: the extent to which adhesion molecules at the protrusion tip are bound to the substrate
$\frac{dC}{dt} = -\frac{AD}{VL}(C - C_0) + r\frac{V_0}{V} C_0$	Change in the concentration of clutch molecules at the protrusion tip
$\frac{d\mathbf{x}_{cb}}{dt} = \eta \sum_i^n \mathbf{F}_{L,i}(t - dt)$	Change in the position of the cell body $\mathbf{x}_{cb}$: determined by the pulling force of each protrusion
$k_p(M) = k_{pr} \left(\frac{M}{E_m}\right)^4$	Protrusion emergence rate: proportional to the fourth power of the amount of cytoskeletal components [9,10]
$M = E_m - \sum_i^n L_i$	Free cytoskeletal component amount (total length): defined as the total amount of components E_m minus the length of each protrusion L_i
$L(t) = \lvert \mathbf{x}_{tip}(t) - \mathbf{x}_{cb}(t) \rvert$	Length of protrusion i : defined as the distance from the cell body center to the protrusion tip

Table H. Statistical test results and JS divergences from 16 independent fitting runs.
Parameter estimation was performed in 16 independent runs. In each run, all model parameters were first estimated from the experimentally observed distributions under the fibronectin condition. Using these estimates as a reference, only the bound-fraction parameters were subsequently estimated from the distributions under the laminin condition. For each run, differences between the two ECM conditions were tested for five motility-related features (turning angle, speed, protrusion number, maximum protrusion length, and protrusion lifetime) using mixed-effects models with ECM condition as a fixed effect and cell identity as a random effect. Statistical significance is indicated as follows: *$p < 0.05$; **$p < 0.01$; ***$p < 0.001$; n.s., not significant. The summed Jensen–Shannon divergences between the model-predicted and experimentally observed distributions across the five features are also shown (JS div.). For example, the "Lam" column under "JS div. (Fib)" represents the summed JS divergences between the distributions predicted using the fibronectin-fitted parameter set and the experimentally observed distributions under the laminin condition. Reproduction of an experimentally observed ECM-dependent difference required both statistical significance and the same direction of effect as in the experimental data. Shaded cells indicate cases in which either (i) the significance or direction of the ECM-dependent difference did not match the experimental results shown in **Fig 1F** or (ii) the summed JS divergence was larger for the matched model–experiment comparison than for the corresponding mismatched comparison. The dagger (†) indicates the parameter set used for the results presented in the main text.

	Turning angle	Speed	Number	Max length	Lifetime	JS div. (Fib)		JS div. (Lam)	
						Fib	Lam	Fib	Lam
1	*	n.s.	***	***	***	0.17	0.35	0.19	0.26
2†	***	***	***	***	***	0.14	0.27	0.33	0.17
3	*	***	***	***	***	0.16	0.32	0.23	0.21
4	n.s.	***	***	***	***	0.21	0.29	0.33	0.22
5	***	***	***	***	***	0.17	0.38	0.28	0.29
6	n.s.	***	***	***	***	0.22	0.28	0.36	0.18
7	n.s.	***	***	***	***	0.15	0.32	0.28	0.20
8	*	n.s.	***	***	***	0.21	0.40	0.22	0.26
9	***	***	***	***	***	0.17	0.32	0.23	0.25
10	n.s.	***	***	***	*	0.15	0.24	0.22	0.18
11	n.s.	***	***	***	***	0.16	0.36	0.25	0.22
12	n.s.	***	***	***	***	0.17	0.34	0.29	0.24
13	*	***	***	***	***	0.18	0.32	0.30	0.29
14	*	***	***	***	***	0.14	0.28	0.18	0.22
15	***	***	***	***	***	0.22	0.35	0.36	0.19
16	n.s.	***	***	***	***	0.21	0.37	0.26	0.26

Table I. Statistical significance across 100 random cell-subsampling iterations.
Summary of the statistical results across 100 iterations. In each iteration, five of the 10 simulated cells were randomly selected without replacement from each ECM condition. Differences between the fibronectin and laminin conditions were tested using mixed-effects models with ECM condition as a fixed effect and cell identity as a random effect. Statistical significance was defined as $p < 0.05$.

Motility-related feature	Significant, n (%)	Not significant, n (%)
Turning angle	97 (97%)	3 (3%)
Speed	100 (100%)	0 (0%)
Protrusion number	100 (100%)	0 (0%)
Maximum protrusion length	100 (100%)	0 (0%)
Protrusion lifetime	100 (100%)	0 (0%)
All five features	97 (97%)	3 (3%)

Movie Legends

S1 Movie (separate file). Cell migration on fibronectin
GDC40 migration on a 2D plane on a fibronectin-coated matrix. The matrix concentration gradient was uniform.

S2 Movie (separate file). Cell migration on laminin
GDC40 migration on a 2D plane on a laminin-coated matrix. The matrix concentration gradient was uniform.

S3 Movie (separate file). Cell migration in simulation (F-ECM condition)
Model simulation video assuming cell migration on fibronectin. Parameters estimated from images of GDC40 cell migration on fibronectin were used (F-ECM condition).

S4 Movie (separate file). Cell migration in simulation (L-ECM condition)
Model simulation video assuming cell migration on laminin. Parameters estimated from images of GDC40 cell migration on laminin were used (L-ECM condition).

SI References

1. Birdal T. Maximum inscribed circle using Voronoi diagram. In: MATLAB Central File Exchange. [Internet]. [cited 6 Dec 2022]. Available: https://www.mathworks.com/matlabcentral/fileexchange/32543-maximum-inscribed-circle-using-voronoi-diagram

2. Toriyama M, Sakumura Y, Shimada T, Ishii S, Inagaki N. A diffusion-based neurite length-sensing mechanism involved in neuronal symmetry breaking. Mol Syst Biol. 2010;6: 394. doi:10.1038/msb.2010.51

3. Bell GI. Models for the specific adhesion of cells to cells. Science. 1978;200: 618–627. doi:10.1126/science.347575

4. Zhurkov SN. Kinetic concept of the strength of solids. Int J Fract. 1965;1: 311–323. doi:10.1007/bf03545562

5. Li Y, Bhimalapuram P, Dinner AR. Model for how retrograde actin flow regulates adhesion traction stresses. J Phys: Condens Matter. 2010;22: 194113. doi:10.1088/0953-8984/22/19/194113

6. Bangasser BL, Rosenfeld SS, Odde DJ. Determinants of maximal force transmission in a motor-clutch model of cell traction in a compliant microenvironment. Biophys J. 2013;105: 581–592. doi:10.1016/j.bpj.2013.06.027

7. Freedman D, Diaconis P. On the histogram as a density estimator: L2 theory. Z für Wahrscheinlichkeitstheorie Verwandte Geb. 1981;57: 453–476. doi:10.1007/bf01025868

8. Razavi N. Jensen-Shannon divergence. In: MATLAB Central File Exchange [Internet]. [cited 2 Sept 2023]. Available: https://www.mathworks.com/matlabcentral/fileexchange/20689-jensen-shannon-divergence

9. Kasai M, Asakura S, Oosawa F. The cooperative nature of G-F transformation of actin. Biochim Biophys Acta. 1962;57: 22–31. doi:10.1016/0006-3002(62)91073-9

10. Tobacman LS, Korn ED. The kinetics of actin nucleation and polymerization. J Biol Chem. 1983;258: 3207–3214. doi:10.1016/s0021-9258(18)32850-3
