# Supplementary material for: Mechanical polarity links adhesion-regulated protrusions to directional stability in glioblastoma cell migration
Source: PLoS Comput Biol. 2026 Sep 24;22(9):e1014772. doi: 10.1371/journal.pcbi.1014772 (PMC13606977; doi:10.1371/journal.pcbi.1014772)
Supplement: Supplementary file 3 [file pcbi.1014772.s008.docx]

Andreas Buttenschoen	July 27, 2026

Academic Editor
PLOS Computational Biology

Dimitrios Vavylonis
Section Editor
PLOS Computational Biology

**Re: Revised manuscript PCOMPBIOL-D-26-00855**

**Original title:** “Mechanical polarity governs directional stability through adhesion-regulated protrusions in glioblastoma cell migration”
**Revised title:** “Mechanical polarity links adhesion-regulated protrusions to directional stability in glioblastoma cell migration”

Dear Drs. Buttenschoen and Vavylonis,

Thank you for the opportunity to revise our manuscript. We have carefully considered all comments from the editors and reviewers and have revised the manuscript accordingly.

The major revisions include additional analyses of the relationships between mechanical polarity and migration dynamics, the use of mixed-effects models to account for repeated observations within individual cells, analyses of temporal-resolution and sample-size effects, and repeated parameter-fitting runs to evaluate the robustness of the parameter estimates. We have also expanded the descriptions of the model formulation, numerical implementation, optimization procedure, and parameter estimation, and have clarified the limitations concerning parameter sensitivity and identifiability.

In addition, we changed the manuscript title as shown above. The revised title uses “links” rather than “governs” to more accurately reflect the scope of our findings and to avoid implying a stronger causal or generalizable conclusion than is supported by the present study.

A detailed point-by-point response to the reviewers’ comments is provided in the accompanying Response to Reviewers. All changes are indicated in the tracked-changes version of the manuscript (“Revised Manuscript with Track Changes.docx”), and a clean version without tracked changes (“Manuscript.docx”) is also provided. We have also made corrections to the appendix (“S1 Appendix.pdf”, formerly “Supplement.pdf”).

We sincerely appreciate the constructive comments from the editors and reviewers, which have helped us substantially improve the manuscript. We hope that the revised manuscript is now suitable for publication in PLOS Computational Biology.

Sincerely,

Yonehiro Kanemura and Yuichi Sakumura
Corresponding Authors
On behalf of all authors

**Point-by-point response to Reviewer #1:**

We sincerely thank Reviewer #1 for the careful reading of our manuscript and for the constructive and encouraging comments. We have revised the manuscript accordingly and respond to each point below.

*Major comments
1. The authors claim that mechanical polarity is a good indicator of migration efficiency. This claim appears to be based mainly on statistical differences between two (plus one) parameter sets corresponding to cells on fibronectin and laminin (plus the parameters used in Fig. 4). Given that cell behaviors show substantial fluctuations even under a single condition, it should be possible to test more directly whether mechanical polarity correlates with dynamical indicators of migration (e.g., instantaneous speed, persistence, turning angle) within a single sufficiently long simulated trajectory. Please quantify such correlations. This would provide more direct support for the proposed role of mechanical polarity.*

Thank you for this important suggestion. To address this point, we performed an additional within-trajectory analysis for each simulated cell under both ECM conditions. Mechanical polarity, migration speed, and turning angle were calculated at 2-minute intervals along the same 600-minute trajectories, and their relationships were evaluated using Spearman’s correlation coefficient. The results showed that mechanical polarity correlated moderately positively with speed (r = 0.4 to 0.6) and weakly negatively with the turning angle (r = − 0.3 to − 0.1) (**Fig. S4 and S5**). Thus, mechanical polarity was consistently associated with more efficient instantaneous displacement, whereas its association with directional change was weaker. The weaker association with turning angle may reflect the fact that turning angle depends on temporal changes in migration direction, whereas mechanical polarity was evaluated as an instantaneous force-alignment measure. We have added these analyses to the revised manuscript in Results p.6, lines 285-294 and S1 Appendix, **Fig. S4 and S5**.

*2. How can mechanical polarity be measured experimentally? Please discuss feasible approaches. For example, traction force microscopy could be used to estimate force distributions and their asymmetry, which may provide an experimental proxy for mechanical polarity.*

We thank the reviewer for this helpful suggestion. In the revised Discussion, we added a paragraph describing possible experimental approaches for mechanical polarity. In particular, we now note that traction force microscopy could provide spatial distributions of traction forces and thereby enable estimation of force asymmetry and net-force alignment across protrusions. We also discuss that protrusion orientation and local adhesion dynamics, if combined with force measurements or suitable live-cell imaging of adhesion markers, may provide an indirect experimental approximation of the same concept. While mechanical polarity was not directly measured in the present study, we now clarify that it may be experimentally approximated through force-distribution-based approaches, including traction force microscopy. This discussion has been added to Discussion, page 9-10, lines 489–497.

*3. Because the model is relatively complex and contains many parameters, it would be helpful to assess the sensitivity of the key conclusions to parameter choice. I understand that parameters are selected by best fitting the experimental data (e.g., via JS divergence), but please confirm that the main qualitative results remain unchanged under reasonable perturbations of the fitted parameters.*

Thank you for this important comment. To examine whether the main conclusions depended on a particular fitted parameter set, we performed 16 independent fitting runs starting from the same initial parameter values (**Fig. S2**). Owing to the stochastic nature of the fitting procedure, these runs converged to distinct parameter combinations, with several fitted values differing by more than 10% from their across-run means.

Despite this variability in the fitted parameters, the experimentally observed ECM-dependent phenotypic pattern was consistently reproduced across the independently obtained solutions. Specifically, among the four features that showed significant differences between the fibronectin and laminin conditions in the experimental data—turning angle, migration speed, protrusion number, and maximum protrusion length—at least three were reproduced in all 16 fitted solutions, and all four were reproduced in 7 of the 16 solutions (**Table S8**).

In addition, the overall agreement between the model-predicted and experimental distributions was assessed using the summed Jensen–Shannon divergence across the five measured features. For the fibronectin-fitted model, the matched model–experiment comparison showed a lower summed JS divergence than the corresponding mismatched comparison in all 16 solutions. For the laminin-fitted model, this relationship was observed in 10 of the 16 solutions.

We also examined correlations among the fitted parameters across the 16 solutions and found that several parameters covaried, suggesting compensatory relationships among them (**Fig. S3**). Because the fitted parameters were not independent, perturbing one parameter at a time while holding all others fixed can generate parameter combinations that were not represented among the solutions supported by the fitting procedure. We therefore used the ensemble of independently fitted parameter sets to assess robustness across fitted parameter combinations while retaining these compensatory relationships.

These results indicate that, although the individual fitted parameter values are not unique, the overall ECM-dependent phenotypic pattern was relatively robust across distinct fitted parameter combinations. However, complete reproduction of all experimentally observed differences was not achieved in every solution, and the distribution-level agreement was less consistent for the laminin-fitted model. We have added the parameter distributions and correlations to **Fig. S2 and S3**, respectively, and the corresponding phenotypic and distribution-level comparisons to Table S8. We also clarified in the manuscript that the conclusions are supported by an ensemble of fitted solutions rather than by a single parameter set (S1 Appendix, **Fig. S2 and S3**, **Table S8**; Results, page 4, lines 200–215).

*Minor comments
1. It would be helpful to show representative migration trajectories for both experiments (D- and L-GDC40) and simulations (for each parameter set). I would prefer moving Fig. S1 to the main text.*

Thank you for this suggestion. We moved the representative migration trajectories previously shown in **Fig. S1** to the main text as new **Fig. 3**, so that both the experimental and simulated trajectory patterns can be directly compared in the main Results section.

*2. In several figures, the authors use the Wilcoxon rank-sum test. However, given the sufficiently large sample sizes, I do not think that small p-values are necessarily informative. Moreover, some sample points may be derived from the same cells and strict independence may not hold. Although I think there is no serious problem with the authors’ conclusions (since violin plots are provided), I think there is room to improve the statistical presentation. There are several possible ways to address this issue (using hierarchical models and/or reporting effect sizes such as the rank-biserial correlation). I encourage the authors to consider these options and include the additional analyses in the Supplementary Information.*

We appreciate this careful comment. To address this concern, we performed mixed-effects analyses with ECM condition as a fixed effect and cell identity as a random effect to account for within-cell clustering of repeated measurements. Since Reviewer #3 also pointed out the issue of statistical independence, and we recognized this as an important point, we replaced all results from the Wilcoxon rank-sum test with those from the mixed-model analysis, rather than simply adding them to the appendix. Importantly, the overall trends remained consistent with the original conclusions, although some significance levels changed. We have revised the text to present the additional statistics more appropriately (Results, page 6, lines 280, 307–308; Methods, page 13-14, lines 693–702; **Fig. 1** and its caption; **Fig. 5** and its caption; the caption for **Fig. S1**; **Fig. S6** and its caption).

In addition to the changes described above, we also corrected typographical errors, figure and equation references, and minor wording issues throughout the manuscript and Supporting Information.

**Point-by-point response to Reviewer #2:**

We sincerely thank Reviewer #2 for the positive evaluation of our study and for the constructive suggestions. We have revised the manuscript accordingly and respond to each comment below.

*1) It is hard to understand qualitatively how the model works. If I understand correctly, one important factor is limited tubulin resource, another is length of protrusion effect on the adhesion delivery to the tip, but the principal feedbacks are elusive. For now, the model reads like: here are the equations, and simulations gave us these results. But why? Give readers some intuition how all this works.*

Thank you for this important comment. We have revised the manuscript to explain more clearly the principal feedback mechanisms underlying the simulations.

At the protrusion level, protrusion length affects the accumulation of clutch molecules, $C$, at the protrusion tip. This process is represented by the Fick’s-law-based diffusion term in $dC/dt$ (Eq. 10). As protrusions become longer, diffusive redistribution from the protrusion tip toward the cell body becomes slower, thereby promoting the accumulation of $C$ at the tip. The accumulated clutch molecules enhance the generation of protrusive force ($F_{E}$; Eq. 6), and this force feeds back to protrusion elongation through the second term in the equation for $v_{tip}$ (Eq. 4).

Once $F_{E}$ enters the high-force regime in which the slip-bond component dominates, further increases in force promote adhesion detachment and reduce the bound fraction ($g$; Eq. 7). In addition, when protrusions become sufficiently long, the tension $F_{L}$ (Eq. 5) increases, and this force also acts through $v_{tip}$ (Eq. 4) to promote protrusion retraction. Thus, protrusion length, clutch molecule accumulation, protrusive force, adhesion detachment, and tension together form feedback loops that regulate protrusion elongation and retraction.

At the whole-cell level, multiple protrusions compete for limited cytoskeletal resources ($M$; Eq. 9) and clutch molecules $C$. Therefore, frequent protrusion emergence distributes the available resources among many protrusions, leading to shorter and less stable protrusions. In contrast, the maintenance of fewer stable protrusions promotes spatially biased force generation, mechanical polarity, and persistent cell migration.

We have added this explanation to the manuscript to provide a more intuitive understanding of how the model behaves (Results, page 3-4, lines 151–166).

*2) "ECM composition modulates migration by altering effective adhesion" - how could ECM affect the diffusion??? and then: "These ECM-dependent differences ... arise from changes in adhesion stability" So what is it? Stability? Diffusion?*

Thank you for pointing out this ambiguity. Our intention was not to claim that ECM composition alters the diffusion itself. Rather, in our model, ECM-dependent effects are represented through changes in effective adhesion-related parameters, which influence the stability of protrusion–substrate interactions. As adhesions become more stable, protrusions are maintained and extended more easily. Because the diffusive return term decreases with protrusion length, clutch molecules are more likely to remain near the protrusion tips. Thus, although this may appear as reduced diffusion toward the cell body, the primary cause in our model is adhesion stabilization, not a direct change in diffusion. We have added a paragraph to make this point clearer. We have revised the relevant text in Discussion, page 7, lines 359–363.

*3) Line 134: "Protrusions emerge stochastically (ii) and are retracted and removed when their length falls below a defined threshold" - I do not understand what this means and do not see anything like that in the equations...*

Thank you for highlighting this lack of clarity. The protrusion emergence and protrusion removal are implemented as explicit update rules in the simulation procedure, rather than as continuous terms written directly in the main differential equations. The timing of protrusion initiation is determined based on the probability calculated in Equation 8. The protrusion is removed from the simulation when its length falls to or below the threshold ($L_{0}$). These update rules are described in the Methods (page 12, lines 615-626). We have revised the text of the Results and Methods to make this distinction clearer (Results, page 3, lines 139–141, and Methods, page 12, lines 625–626).

*4) Line 190: "The estimated ratios of the four adhesion-related parameters ( , , , ) between the F-ECM and L-ECM conditions were 1.07, 1.38, 0.91, and 1.17, respectively." - these are relatively small changes suggesting that the model is not very robust. Comment on what this means for the migratory behavior when a small changes of the parameters lead to such significant change in phenotype? How could this work considering a usual order of magnitude fluctuation of cell parameters.*

Thank you for this important comment. Although the fitted adhesion-related parameters differed only modestly between the F-ECM and L-ECM conditions, their effects accumulate over time through the model dynamics. At each time step, these parameters influence the stability of protrusion–substrate adhesion and therefore the balance between protrusion maintenance, elongation, and retraction. Even a small difference in this balance can be repeatedly integrated over the course of the simulation, leading to progressively different protrusion configurations and force coordination. As a result, modest parameter differences can produce substantial differences in time-evolving migration phenotypes, including migration speed and directional persistence. We have clarified this point in the revised Discussion, page 8, lines 397–404.

*5) Bottom row of Fig 5 is confusing and not informative: why would the protrusion length go down if the protrusion rate is up?*

Thank you for this important comment. First, the term “protrusion rate” refers not to the speed at which protrusions extend, but to the frequency with which they emerge. To make this clearer, we have replaced this term in the revised manuscript with “protrusion emergence rate.” Therefore, increasing the protrusion emergence rate does not necessarily increase protrusion length, because more frequent protrusion initiation increases competition for limited cytoskeletal and clutch molecules. As a result, individual protrusions are maintained less stably and tend to remain shorter on average, even though protrusion formation events become more frequent. We revised the text and the figure description accordingly to make this mechanism more intuitive. We have updated the explanation in the Discussion (page 8, lines 407–411), and **Fig. 6**.

In addition to the changes described above, we also corrected typographical errors, figure and equation references, and minor wording issues throughout the manuscript and Supporting Information.

**Point-by-point response to Reviewer #3:**

We sincerely thank Reviewer #3 for the careful reading of our manuscript and for the thoughtful and constructive comments. We have carefully revised the manuscript in response to the points raised and provide our detailed responses below.

*1. Positioning relative to prior modeling literature. The introduction does not adequately discuss prior published models. A few papers to consider: Holmes et al. (2017, https://doi.org/10.1371/journal.pcbi.1005524) generates similar phenotypic regimes through Rac–Rho signaling competition, and Hou et al. (2024, https://doi.org/10.1093/noajnl/vdae184) is a relevant glioblastoma-specific motor-clutch work.*

Thank you for this important suggestion. In the revised manuscript, we added a paragraph that more clearly places our model in the context of previous approaches, including the signaling-centered polarity model of Holmes et al. and the glioblastoma-specific motor-clutch framework of Hou et al. We clarify that our model differs in its level of abstraction: rather than explicitly modeling full intracellular signaling networks or detailed molecular clutch machinery, we focus on protrusion-level force generation and effective force-dependent adhesion kinetics in order to quantify how force coordination governs migration. This revision better distinguishes our approach from both signaling-centered polarity models and more detailed motor-clutch frameworks. These changes have been incorporated in the revised Introduction, page 2, lines 74–85.

*2. Temporal resolution of speed and turning angle measurements. Speed is computed over 2-minute intervals while turning angle is computed over 4-minute intervals. This inconsistency is concerning because speed and persistence are coupled in cell migration (Maiuri et al. 2015, https://doi.org/10.1016/j.cell.2015.01.056), and using 4-minute resolution for turning angle may smooth out directional correlations.*

Thank you for this important comment. Our primary reason for using a 4-minute interval for turning angle was that directional estimates are especially sensitive to short-timescale fluctuations when the displacement over a single interval is small. Because displacement vectors are shorter at 2-minute intervals than at 4-minute intervals (**Fig. S8E,F**), turning-angle estimates at the shorter interval are more susceptible to small shifts in cell-body coordinates and other short-timescale noise. In contrast, speed depends on displacement magnitude rather than direction and is therefore less sensitive to this specific source of noise. For this reason, we retained the 2-minute definition for speed while using a 4-minute interval for turning angle.

To confirm that the main conclusions remain valid even when turning angle is measured at 2-minute intervals, we recalculated turning-angle distributions using 2-minute intervals (**Fig. S8A-D**). In the experimental data, the turning-angle distributions were broadly similar between the 2-minute and 4-minute definitions, indicating that the experimentally observed ECM-dependent difference in directional stability was not strongly dependent on this choice. In the simulations, however, the fibronectin condition showed an excess of small turning angles at 2-minute resolution, indicating that the current model overestimates short-timescale directional persistence under this condition.

We added text to the Discussion noting that the mismatch at 2-minute resolution suggests that the current model does not fully capture the rapid directional fluctuations observed experimentally, particularly under the fibronectin condition. Importantly, the main qualitative conclusion that laminin promotes more directionally stable migration than fibronectin remains unchanged.

These revisions are included in Discussion, page 9, lines 467-480; Methods, page 10, lines 529-535 and S1 Appendix, **Fig. S8**.

*3. Sample size and statistical independence. Five cells per ECM condition is a very small sample. Although the n in Figure 1F appears adequate, these data points come from only 5 cells, so the possibility that within-cell measurements are dependent is high. The Wilcoxon rank-sum test requires independent samples between the two groups, but also within each group, and in the present design this independence has not been established. I suggest that the authors expand their dataset to more than 5 cells and establish statistical independence.*

We thank the reviewer for this careful comment and agree that the original statistical presentation did not sufficiently address the non-independence of repeated measurements from the same cell. Although it was not feasible to expand the experimental dataset within the scope of the present revision, we addressed this concern as follows. We performed mixed-effects analyses with ECM condition as a fixed effect and cell identity as a random effect to account for within-cell clustering of repeated measurements. These additional analyses showed that the overall trends remained consistent with the original conclusions, while making the statistical treatment more appropriate for the nested structure of the data.

Because Reviewer #1 raised the same concern regarding statistical independence, and because we recognized this as an important point, we replaced all results from the Wilcoxon rank-sum test with those from the mixed-model analysis. These changes are included in Results, page 6, lines 280, 307–308; Methods, page 13-14, lines 693–702; **Fig. 1** and its caption; **Fig. 5** and its caption; the caption for **Fig. S1**; **Fig. S6** and its caption.

We acknowledge that this reanalysis does not increase the number of independent biological units and that the limited number of cells remains a limitation of the study. Accordingly, we have softened the scope of our conclusions and now frame them specifically in the context of the GDC40 dataset. We have also added a brief statement to the Discussion (page 9, lines 454–457) noting that this limitation remains despite the use of mixed-effects models.

*4. Fitting procedure is underspecified. The full fitting process should be stated explicitly. The optimization algorithm should be named. Moreover, the exact form of the objective function, and any relevant hyperparameter values should all be reported. The numerical method used to solve the ODEs is also not specified. A table listing every parameter, its meaning, its value, whether it was fixed or fitted at each stage, and the source of fixed values would substantially improve reproducibility.*

Thank you for this important and constructive comment. In the revised manuscript, we now clarify that parameter estimation was performed using a custom stochastic local search procedure implemented in MATLAB, rather than a standard built-in optimization routine (Methods, page 13, lines 673–681). Briefly, starting from an initial parameter set, candidate parameter values were generated by adding Gaussian perturbations to the current parameter values. For each candidate set, the model was simulated and the objective function was evaluated as the sum of the Jensen–Shannon (JS) divergences between the simulated and experimental distributions of the migration/protrusion features (Methods, page 13, lines 659-662). Candidate parameters were accepted only when they decreased the objective value; otherwise, the previous parameter set was retained. This procedure was repeated until no improvement in the objective value was observed for 500 consecutive proposal steps, and the final retained parameter set was taken as the estimated solution. The details on how to calculate JS divergence are also described in S1 Appendix, **Text S4**.

We also revised the manuscript to state the numerical solver explicitly (Methods, page 13, lines 689–690). The continuous model variables were updated using the Euler method with a time step of *Δt* = 0.01, while protrusion appearance and removal were implemented as discrete update rules during the simulation.

We revised the supplementary table (S1 Appendix, **Table S5**) so that it now includes not only the meaning, units, numerical values, and sources or rationale for fixed parameter values, but also an indication of whether each parameter was treated as fixed or estimated at each fitting stage.

*5. Sensitivity analysis and parameter identifiability. In the second stage of the fitting process, only a subset of four parameters (a_s, b_s, a_c, b_c) is allowed to vary. A formal sensitivity analysis showing that the model output is more sensitive to these four than to the other parameters held fixed would substantially strengthen the claim that these parameters are the relevant degrees of freedom. Furthermore, with 19 free parameters fitted against five feature distributions from five cells, some parameters are likely to be non-identifiable. An identifiability analysis would also strengthen the manuscript.*

Thank you for this important comment. To examine practical identifiability and the robustness of the principal conclusions across alternative fitted solutions, we performed 16 independent fitting runs starting from the same initial parameter values. Owing to the stochastic nature of the fitting procedure, these runs converged to distinct parameter combinations, with several fitted values differing by more than 10% from their across-run means (**Fig. S2**).

Despite this variability, at least three of the four experimentally observed ECM-dependent differences—turning angle, migration speed, protrusion number, and maximum protrusion length—were reproduced in all 16 fitted solutions, and all four were reproduced in 7 of the 16 solutions (**Table S8**). In addition, the matched model–experiment comparison showed a lower summed Jensen–Shannon divergence than the corresponding mismatched comparison in all 16 solutions for the fibronectin-fitted model and in 10 of 16 solutions for the laminin-fitted model.

We also examined correlations among the fitted parameters across the 16 solutions and found that several parameters covaried, suggesting compensatory relationships among them (**Fig. S3**). These results indicate that the available data do not uniquely constrain the individual fitted parameters and that the present fitting framework therefore has limited practical identifiability.

Because the fitted parameters were not independent, perturbing one parameter at a time while holding all others fixed could generate parameter combinations that were not represented among the fitted solutions. We therefore used the ensemble of independently fitted parameter sets to assess robustness across parameter combinations supported by the fitting procedure while retaining compensatory relationships among parameters. This ensemble analysis is not a formal structural identifiability analysis, but it directly assesses whether the main ECM-dependent phenotypic interpretation depends on a single fitted parameter set.

As noted in the Discussion (p. 7, lines 346-351), our intention in the second fitting stage was not to demonstrate the uniqueness of these four parameters. Rather, our aim was to test a more specific modeling hypothesis: that the ECM-dependent differences can be explained within a framework in which ECM acts primarily through adhesion mechanics, without directly altering intracellular processes. Based on this hypothesis, we intentionally restricted ECM dependence to the adhesion-related parameters. From this perspective, the main result of this analysis is not that these parameters are the only possible explanation, but that they are sufficient to reproduce the observed ECM-dependent differences under this modeling assumption.

We have added the parameter distributions and correlations to **Fig. S2 and S3**, respectively, and the phenotypic and distribution-level comparisons across the 16 fitted solutions to **Table S8**. We also clarified in the revised manuscript that the individual parameter values are not uniquely identifiable, whereas the overall ECM-dependent phenotypic pattern is relatively robust across distinct fitted parameter combinations (Results, page 4, lines 200–215).

*6. Generalization claims based on a single cell line. The Discussion concludes that “this principle likely extends beyond glioblastoma biology, providing a generalizable mechanism.” The support is from one patient-derived cell line (GDC40), two ECM proteins at one concentration each, and five cells per condition. The authors should soften the generalization language and frame the conclusions as hypotheses applicable to GDC40 specifically.*

Thank you for this important comment. We agree that the original wording exceeded the scope supported by a single patient-derived cell line, two ECM conditions, and a limited number of cells. We have therefore softened the generalization language throughout the manuscript. Mechanical polarity is now described as a candidate physical descriptor or principle supported in the GDC40 system, rather than as an established general mechanism across cell types. These revisions are reflected in Abstract (page 1, lines 29–32), Introduction (page 2, lines 100–101), Discussion (page 8, lines 415–419).

*7. Lifetime discrepancy between data and model. Protrusion lifetime is statistically indistinguishable between FN and LN in the experimental data (Fig. 1F), but the model produces a significant difference (Fig. 1G). This is the only feature where the model and data diverge. The authors should address this discrepancy in the Results and Discussion sections.*

Thank you for pointing out this discrepancy. We agree that protrusion lifetime is the feature least well reproduced by the current model. The model predicts an ECM-dependent difference, whereas the experimental data do not show a statistically significant difference. This may indicate that the coarse-grained adhesion formulation overestimates how strongly ECM-dependent adhesion differences are translated into protrusion lifetime. The limited experimental sample size may also contribute to the uncertainty. However, we do not regard it as sufficient to dismiss the discrepancy. These revisions are reflected in Results page 4, lines 180-182, and Discussion, page 9, lines 443–452.

*8. Mismatched simulation and experimental sample sizes. Simulations include 10 cells per condition while experiments include 5; the simulated sample size should match the experimental one so that finite-sample effects on the JS divergence are equivalent.*

Thank you for this helpful comment. In our fitting procedure, both simulated and experimental histograms were normalized to unit area before calculating the JS divergence. Therefore, the metric itself does not directly depend on the absolute sample size, and the use of 10 simulated cells versus 5 experimental cells does not by itself bias the divergence through unequal normalization.

To examine the effect of cell number, we performed an additional subsampling analysis in which five cells were randomly selected from the ten simulated cells (100 times). In each of 100 iterations, five of the ten simulated cells were randomly selected without replacement from each ECM condition, and the corresponding feature distributions were recalculated. **Figure S7** shows one representative iteration, whereas **Table S9** summarizes the results across all 100 iterations. Statistically significant ECM-dependent differences in all five features were observed in 97 of the 100 iterations (S1 Appendix, **Table S9**). This includes the protrusion-lifetime difference that was not observed experimentally. The resulting distributions were similar to those obtained from all ten simulated cells (S1 Appendix, **Fig. S7B and C**), and the ECM-dependent differences in motility-related features were also mostly preserved (S1 Appendix, **Fig. S7A**). Thus, the subsampling analysis suggests that the mismatch in cell number did not qualitatively alter the main conclusions.

However, we agree that finite-sample variability in the estimated distributions may still depend on sample size. We have therefore clarified this point in the revised manuscript (S1 Appendix, **Text S4**, page 5, lines 165-178).

*9. Choice of divergence metric. The authors use Jensen-Shannon divergence summed across five features as the fitting objective. The authors should explain why they chose the JS metric instead of others, for example Wasserstein distance or Kullback-Leibler (KL) divergence.*

We thank the reviewer for this helpful comment. We chose the Jensen–Shannon divergence because the fitting was based on histogram distributions evaluated on a common set of bins, and we sought a symmetric measure of differences in probability mass between experimental and simulated distributions. Unlike the Kullback–Leibler divergence, the JS divergence is symmetric and remains finite when the two distributions have non-overlapping support, making it more stable for sparsely populated histogram bins.

We also considered the Wasserstein distance. However, Wasserstein distance explicitly incorporates the metric distance between bins and therefore emphasizes how far probability mass must be transported along the variable axis. In the present study, we prioritized differences in the relative probability assigned to the observed histogram bins, particularly for the bounded and potentially multimodal turning-angle distributions. We have clarified this rationale in the revised Methods, page 13, lines 662–671.

In addition to the changes described above, we also corrected typographical errors, figure and equation references, and minor wording issues throughout the manuscript and Supporting Information.
