# Supplementary material for: Mechanical polarity links adhesion-regulated protrusions to directional stability in glioblastoma cell migration
Source: PLoS Comput Biol. 2026 Sep 24;22(9):e1014772. doi: 10.1371/journal.pcbi.1014772 (PMC13606977; doi:10.1371/journal.pcbi.1014772)
Supplement: Supplementary file 4 [file pcbi.1014772.s006.docx]

The manuscript proposes mechanical polarity as a quantitative scalar that links adhesion mechanics to directional stability in glioblastoma migration. This is a genuine conceptual contribution, and the demonstration that ECM-dependent phenotypes can be reproduced by tuning only four catch/slip parameters cleanly illustrates how adhesion biophysics alone may suffice to account for FN-vs-LN differences. The points below are intended to strengthen the paper before publication.

**Major concerns**

**1. Positioning relative to prior modeling literature.** The introduction does not adequately discuss prior published models. A few papers to consider: Holmes et al. (2017, https://doi.org/10.1371/journal.pcbi.1005524) generates similar phenotypic regimes through Rac–Rho signaling competition, and Hou et al. (2024, https://doi.org/10.1093/noajnl/vdae184) is a relevant glioblastoma-specific motor-clutch work.

**2. Temporal resolution of speed and turning angle measurements.** Speed is computed over 2-minute intervals while turning angle is computed over 4-minute intervals. This inconsistency is concerning because speed and persistence are coupled in cell migration (Maiuri et al. 2015, https://doi.org/10.1016/j.cell.2015.01.056), and using 4-minute resolution for turning angle may smooth out directional correlations.

**3. Sample size and statistical independence.** Five cells per ECM condition is a very small sample. Although the n in Figure 1F appears adequate, these data points come from only 5 cells, so the possibility that within-cell measurements are dependent is high. The Wilcoxon rank-sum test requires independent samples between the two groups, but also within each group, and in the present design this independence has not been established. I suggest that the authors expand their dataset to more than 5 cells and establish statistical independence.

**4. Fitting procedure is underspecified.** The full fitting process should be stated explicitly. The optimization algorithm should be named. Moreover, the exact form of the objective function, and any relevant hyperparameter values should all be reported. The numerical method used to solve the ODEs is also not specified. A table listing every parameter, its meaning, its value, whether it was fixed or fitted at each stage, and the source of fixed values would substantially improve reproducibility.

**5. Sensitivity analysis and parameter identifiability.** In the second stage of the fitting process, only a subset of four parameters (a_s, b_s, a_c, b_c) is allowed to vary. A formal sensitivity analysis showing that the model output is more sensitive to these four than to the other parameters held fixed would substantially strengthen the claim that these parameters are the relevant degrees of freedom. Furthermore, with 19 free parameters fitted against five feature distributions from five cells, some parameters are likely to be non-identifiable. An identifiability analysis would also strengthen the manuscript.

**6. Generalization claims based on a single cell line.** The Discussion concludes that “this principle likely extends beyond glioblastoma biology, providing a generalizable mechanism.” The support is from one patient-derived cell line (GDC40), two ECM proteins at one concentration each, and five cells per condition. The authors should soften the generalization language and frame the conclusions as hypotheses applicable to GDC40 specifically.

**Minor concerns**

**7. Lifetime discrepancy between data and model.** Protrusion lifetime is statistically indistinguishable between FN and LN in the experimental data (Fig. 1F), but the model produces a significant difference (Fig. 1G). This is the only feature where the model and data diverge. The authors should address this discrepancy in the Results and Discussion sections.

**8. Mismatched simulation and experimental sample sizes.** Simulations include 10 cells per condition while experiments include 5; the simulated sample size should match the experimental one so that finite-sample effects on the JS divergence are equivalent.

**9. Choice of divergence metric.** The authors use Jensen-Shannon divergence summed across five features as the fitting objective. The authors should explain why they chose the JS metric instead of others, for example Wasserstein distance or Kullback-Leibler (KL) divergence.
