# Supplementary material for: Mechanical polarity links adhesion-regulated protrusions to directional stability in glioblastoma cell migration
Source: PLoS Comput Biol. 2026 Sep 24;22(9):e1014772. doi: 10.1371/journal.pcbi.1014772 (PMC13606977; doi:10.1371/journal.pcbi.1014772)
Supplement: Peer review history

# Peer review: Decision Letter 0

**DOI:** 10.1371/journal.pcbi.1014772.r001

**Authors:** Dimitrios Vavylonis, Andreas Buttenschoen

25 May 2026

Mechanical polarity governs directional stability through adhesion-regulated protrusions in glioblastoma cell migration

PLOS Computational Biology

Dear Dr. Sakumura,

Thank you for submitting your manuscript to PLOS Computational Biology. After careful consideration, we feel that it has merit but does not fully meet PLOS Computational Biology's publication criteria as it currently stands. Therefore, we invite you to submit a revised version of the manuscript that addresses the points raised during the review process.

Please submit your revised manuscript by Jul 25 2026 11:59PM. If you will need more time than this to complete your revisions, please reply to this message or contact the journal office at ploscompbiol@plos.org. When you're ready to submit your revision, log on to https://www.editorialmanager.com/pcompbiol/ and select the 'Submissions Needing Revision' folder to locate your manuscript file.

Please include the following items when submitting your revised manuscript:

* A letter that responds to each point raised by the editor and reviewer(s). You should upload this letter as a separate file labeled 'Response to Reviewers'. This file does not need to include responses to formatting updates and technical items listed in the 'Journal Requirements' section below.

* A marked-up copy of your manuscript that highlights changes made to the original version. You should upload this as a separate file labeled 'Revised Manuscript with Track Changes'.

* An unmarked version of your revised paper without tracked changes. You should upload this as a separate file labeled 'Manuscript'.

As the corresponding author, your ORCID iD is verified in the submission system and will appear in the published article. PLOS supports the use of ORCID, and we encourage all coauthors to register for an ORCID iD and use it as well. Please encourage your coauthors to verify their ORCID iD within the submission system before final acceptance, as unverified ORCID iDs will not appear in the published article. Only the individual author can complete the verification step; PLOS staff cannot verify ORCID iDs on behalf of authors.

We look forward to receiving your revised manuscript.

Kind regards,

Andreas Buttenschoen

Academic Editor

PLOS Computational Biology

Dimitrios Vavylonis

Section Editor

PLOS Computational Biology

**Journal Requirements:**

If the reviewer comments include a recommendation to cite specific previously published works, please review and evaluate these publications to determine whether they are relevant and should be cited. There is no requirement to cite these works unless the editor has indicated otherwise.

1) We ask that a manuscript source file is provided at Revision. Please upload your manuscript file as a .doc, .docx, .rtf or .tex. If you are providing a .tex file, please upload it under the item type u2018LaTeX Source Fileu2019 and leave your .pdf version as the item type u2018Manuscriptu2019.

2) Please upload all main figures as separate Figure files in .tif or .eps format. For more information about how to convert and format your figure files please see our guidelines:

https://journals.plos.org/ploscompbiol/s/figures

3) We have noticed that you have uploaded Supporting Information files, but you have not included a list of legends. Please add a full list of legends for your Supporting Information files after the references list.

4) In the online submission form, you indicated that These resources will be made publicly available upon acceptance on GitHub. All PLOS journals now require all data underlying the findings described in their manuscript to be freely available to other researchers, either

1. In a public repository

2. Within the manuscript itself

3. Uploaded as supplementary information.

This policy applies to all data except where public deposition would breach compliance with the protocol approved by your research ethics board. If your data cannot be made publicly available for ethical or legal reasons (e.g., public availability would compromise patient privacy), please explain your reasons by return email and your exemption request will be escalated to the editor for approval. Your exemption request will be handled independently and will not hold up the peer review process, but will need to be resolved should your manuscript be accepted for publication. One of the Editorial team will then be in touch if there are any issues.

5) Please amend your detailed Financial Disclosure statement. This is published with the article. It must therefore be completed in full sentences and contain the exact wording you wish to be published.

1) State what role the funders took in the study. If the funders had no role in your study, please state: "The funders had no role in study design, data collection and analysis, decision to publish, or preparation of the manuscript."

2) If any authors received a salary from any of your funders, please state which authors and which funders..

If you did not receive any funding for this study, please simply state: u201cThe authors received no specific funding for this work.u201d

6) Thank you for uploading your study's underlying data set. Unfortunately, the repository you have noted in your Data Availability statement does not qualify as an acceptable data repository according to PLOS's standards.

At this time, please upload the minimal data set necessary to replicate your study's findings to a stable, public repository (such as figshare or Dryad) and provide us with the relevant URLs, DOIs, or accession numbers that may be used to access these data. For a list of recommended repositories and additional information on PLOS standards for data deposition, please see

https://journals.plos.org/ploscompbiol/s/recommended-repositories

**Reviewers' comments:**

Reviewer's Responses to Questions

**Comments to the Authors:**

**Please note here if the review is uploaded as an attachment.**

Reviewer #1: Based on experimental results showing that glioblastoma cells exhibit different migratory behaviors on fibronectin and laminin, the authors propose a cell migration model to explain the difference. They show that, by changing only adhesion-associated parameters (those governing catch- and slip-bond processes), the model can qualitatively reproduce the experimental observations. The authors further propose mechanical polarity as an indicator of migration efficiency, and support this idea with numerical simulations in which the protrusion rate is increased for laminin-like cells.

The data analysis and simulations, including parameter fitting to align the simulations with experimental results, are carefully conducted. The finding that differences in cell behavior on fibronectin versus laminin can be explained solely by changes in adhesion parameters is interesting. Overall, I expect that the manuscript is suitable for publication in PLoS Computational Biology, but I would like to clarify the following points.

Major comments

1. The authors claim that mechanical polarity is a good indicator of migration efficiency. This claim appears to be based mainly on statistical differences between two (plus one) parameter sets corresponding to cells on fibronectin and laminin (plus the parameters used in Fig. 4). Given that cell behaviors show substantial fluctuations even under a single condition, it should be possible to test more directly whether mechanical polarity correlates with dynamical indicators of migration (e.g., instantaneous speed, persistence, turning angle) within a single sufficiently long simulated trajectory. Please quantify such correlations. This would provide more direct support for the proposed role of mechanical polarity.

2. How can mechanical polarity be measured experimentally? Please discuss feasible approaches. For example, traction force microscopy could be used to estimate force distributions and their asymmetry, which may provide an experimental proxy for mechanical polarity.

3. Because the model is relatively complex and contains many parameters, it would be helpful to assess the sensitivity of the key conclusions to parameter choice. I understand that parameters are selected by best fitting the experimental data (e.g., via JS divergence), but please confirm that the main qualitative results remain unchanged under reasonable perturbations of the fitted parameters.

Minor comments

1. It would be helpful to show representative migration trajectories for both experiments (D- and L-GDC40) and simulations (for each parameter set). I would prefer moving Fig. S1 to the main text.

2. In several figures, the authors use the Wilcoxon rank-sum test. However, given the sufficiently large sample sizes, I do not think that small p-values are necessarily informative. Moreover, some sample points may be derived from the same cells and strict independence may not hold. Although I think there is no serious problem with the authors’ conclusions (since violin plots are provided), I think there is room to improve the statistical presentation. There are several possible ways to address this issue (using hierarchical models and/or reporting effect sizes such as the rank-biserial correlation). I encourage the authors to consider these options and include the additional analyses in the Supplementary Information.

Reviewer #2: The authors of this insightful paper introduce a novel quantitative criterion of cell polarity,

simulate cells migrating on ECM by pulling on multiple stochastic protrusions, and conclude

that the differences in migratory behavior between migration on fibronectin and on laminin,

which is quantified in their experiments, can be explained by mechanical differences in adhesion

strength. Another interesting conclusion is that increasing the protrusion emergence rate alone

can disrupt migration efficiency.

The combination of experiment and model is convincing, and the results are important.

I suggest the following minor revisions:

1) It is hard to understand qualitatively how the model works. If I understand correctly,

one important factor is limited tubulin resource, another is length of protrusion effect

on the adhesion delivery to the tip, but the principal feedbacks are elusive. For now, the

model reads like: here are the equations, and simulations gave us these results. But why?

Give readers some intuition how all this works.

2) "ECM composition modulates migration by altering effective adhesion" - how could ECM

affect the diffusion???

and then: "These ECM-dependent differences ... arise from changes in adhesion stability"

So what is it? Stability? Diffusion?

3) Line 134: "Protrusions emerge stochastically (ii) and are retracted and removed when their

length falls below a defined threshold" - I do not understand what this means and do not see

anything like that in the equations...

4) Line 190: "The estimated ratios of the four adhesion-related parameters ( , , , ) between

the F-ECM and L-ECM conditions were 1.07, 1.38, 0.91, and 1.17, respectively." - these are relatively small

changes suggesting that the model is not very robust. Comment on what this means for the

migratory behavior when a small changes of the parameters lead to such significant change in

phenotype? How could this work considering a usual order of magnitude fluctuation of cell

parameters.

5) Bottom row of Fig 5 is confusing and not informative: why would the protrusion length go

down if the protrusion rate is up?

Reviewer #3: The review is also uploaded as an attachment

The manuscript proposes mechanical polarity as a quantitative scalar that links adhesion mechanics to directional stability in glioblastoma migration. This is a genuine conceptual contribution, and the demonstration that ECM-dependent phenotypes can be reproduced by tuning only four catch/slip parameters cleanly illustrates how adhesion biophysics alone may suffice to account for FN-vs-LN differences. The points below are intended to strengthen the paper before publication.

Major concerns

1. Positioning relative to prior modeling literature. The introduction does not adequately discuss prior published models. A few papers to consider: Holmes et al. (2017, https://doi.org/10.1371/journal.pcbi.1005524) generates similar phenotypic regimes through Rac–Rho signaling competition, and Hou et al. (2024, https://doi.org/10.1093/noajnl/vdae184) is a relevant glioblastoma-specific motor-clutch work.

2. Temporal resolution of speed and turning angle measurements. Speed is computed over 2-minute intervals while turning angle is computed over 4-minute intervals. This inconsistency is concerning because speed and persistence are coupled in cell migration (Maiuri et al. 2015, https://doi.org/10.1016/j.cell.2015.01.056), and using 4-minute resolution for turning angle may smooth out directional correlations.

3. Sample size and statistical independence. Five cells per ECM condition is a very small sample. Although the n in Figure 1F appears adequate, these data points come from only 5 cells, so the possibility that within-cell measurements are dependent is high. The Wilcoxon rank-sum test requires independent samples between the two groups, but also within each group, and in the present design this independence has not been established. I suggest that the authors expand their dataset to more than 5 cells and establish statistical independence.

4. Fitting procedure is underspecified. The full fitting process should be stated explicitly. The optimization algorithm should be named. Moreover, the exact form of the objective function, and any relevant hyperparameter values should all be reported. The numerical method used to solve the ODEs is also not specified. A table listing every parameter, its meaning, its value, whether it was fixed or fitted at each stage, and the source of fixed values would substantially improve reproducibility.

5. Sensitivity analysis and parameter identifiability. In the second stage of the fitting process, only a subset of four parameters (a_s, b_s, a_c, b_c) is allowed to vary. A formal sensitivity analysis showing that the model output is more sensitive to these four than to the other parameters held fixed would substantially strengthen the claim that these parameters are the relevant degrees of freedom. Furthermore, with 19 free parameters fitted against five feature distributions from five cells, some parameters are likely to be non-identifiable. An identifiability analysis would also strengthen the manuscript.

6. Generalization claims based on a single cell line. The Discussion concludes that “this principle likely extends beyond glioblastoma biology, providing a generalizable mechanism.” The support is from one patient-derived cell line (GDC40), two ECM proteins at one concentration each, and five cells per condition. The authors should soften the generalization language and frame the conclusions as hypotheses applicable to GDC40 specifically.

Minor concerns

7. Lifetime discrepancy between data and model. Protrusion lifetime is statistically indistinguishable between FN and LN in the experimental data (Fig. 1F), but the model produces a significant difference (Fig. 1G). This is the only feature where the model and data diverge. The authors should address this discrepancy in the Results and Discussion sections.

8. Mismatched simulation and experimental sample sizes. Simulations include 10 cells per condition while experiments include 5; the simulated sample size should match the experimental one so that finite-sample effects on the JS divergence are equivalent.

9. Choice of divergence metric. The authors use Jensen-Shannon divergence summed across five features as the fitting objective. The authors should explain why they chose the JS metric instead of others, for example Wasserstein distance or Kullback-Leibler (KL) divergence.

**********

**Have the authors made all data and (if applicable) computational code underlying the findings in their manuscript fully available?**

The PLOS Data policy requires authors to make all data and code underlying the findings described in their manuscript fully available without restriction, with rare exception (please refer to the Data Availability Statement in the manuscript PDF file). The data and code should be provided as part of the manuscript or its supporting information, or deposited to a public repository. For example, in addition to summary statistics, the data points behind means, medians and variance measures should be available. If there are restrictions on publicly sharing data or code —e.g. participant privacy or use of data from a third party—those must be specified.

Reviewer #1: Yes

Reviewer #2: Yes

Reviewer #3: Yes

**********

PLOS authors have the option to publish the peer review history of their article (what does this mean?). If published, this will include your full peer review and any attached files.

If you choose “no”, your identity will remain anonymous but your review may still be made public.

**Do you want your identity to be public for this peer review?**  For information about this choice, including consent withdrawal, please see our Privacy Policy.

Reviewer #1: No

Reviewer #2: No

Reviewer #3: No

[NOTE: If reviewer comments were submitted as an attachment file, they will be attached to this email and accessible via the submission site. Please log into your account, locate the manuscript record, and check for the action link "View Attachments". If this link does not appear, there are no attachment files.]

**Figure resubmission:**

After uploading your figures to PLOS’s NAAS tool - https://ngplosjournals.pagemajik.ai/artanalysis, NAAS will process the files provided and display the results in the "Uploaded Files" section of the page as the processing is complete. If the uploaded figures meet our requirements (or NAAS is able to fix the files to meet our requirements), the figure will be marked as "fixed" above. If NAAS is unable to fix the files, a red "failed" label will appear above. When NAAS has confirmed that the figure files meet our requirements, please download the file via the download option, and include these NAAS processed figure files when submitting your revised manuscript.

**Reproducibility:**

To enhance the reproducibility of your results, we recommend that authors of applicable studies deposit laboratory protocols in protocols.io, where a protocol can be assigned its own identifier (DOI) such that it can be cited independently in the future. Additionally, PLOS ONE offers an option to publish peer-reviewed clinical study protocols. Read more information on sharing protocols at https://plos.org/protocols?utm_medium=editorial-email&utm_source=authorletters&utm_campaign=protocols

Attachment Submitted filename: Review_plos_one_computational_biology.docx

# Author comment: Author response to Decision Letter 1

**DOI:** 10.1371/journal.pcbi.1014772.r002

28 Jul 2026

Attachment Submitted filename: Response to Reviewers.docx

# Peer review: Decision Letter 1

**DOI:** 10.1371/journal.pcbi.1014772.r003

**Authors:** Dimitrios Vavylonis, Andreas Buttenschoen

28 Aug 2026

Dear Dr. Sakumura,

We are pleased to inform you that your manuscript 'Mechanical polarity links adhesion-regulated protrusions to directional stability in glioblastoma cell migration' has been provisionally accepted for publication in PLOS Computational Biology.

Before your manuscript can be formally accepted you will need to complete some formatting changes, which you will receive in a follow up email. A member of our team will be in touch with a set of requests.

Please note that your manuscript will not be scheduled for publication until you have made the required changes, so a swift response is appreciated.

IMPORTANT: The editorial review process is now complete. PLOS will only permit corrections to spelling, formatting or significant scientific errors from this point onwards. Requests for major changes, or any which affect the scientific understanding of your work, will cause delays to the publication date of your manuscript.

Should you, your institution's press office or the journal office choose to press release your paper, you will automatically be opted out of early publication. We ask that you notify us now if you or your institution is planning to press release the article. All press must be co-ordinated with PLOS.

Thank you again for supporting Open Access publishing; we are looking forward to publishing your work in PLOS Computational Biology.

Best regards,

Andreas Buttenschoen

Academic Editor

PLOS Computational Biology

Dimitrios Vavylonis

Section Editor

PLOS Computational Biology

***********************************************************

Please do look at Reviewer 1s comment regarding one possible clarity comment.

Reviewer's Responses to Questions

**Comments to the Authors:**

**Please note here if the review is uploaded as an attachment.**

Reviewer #1: Regarding the new paragraphs LL. 200-215, I think the results themselves are good, but the flow of the text is somewhat awkward, so it may be better to rewrite this section.

Reviewer #2: I am satisfied with the revisions

Reviewer #3: I would like to thank the authors for revising the manuscript carefully.

**********

**Have the authors made all data and (if applicable) computational code underlying the findings in their manuscript fully available?**

The PLOS Data policy requires authors to make all data and code underlying the findings described in their manuscript fully available without restriction, with rare exception (please refer to the Data Availability Statement in the manuscript PDF file). The data and code should be provided as part of the manuscript or its supporting information, or deposited to a public repository. For example, in addition to summary statistics, the data points behind means, medians and variance measures should be available. If there are restrictions on publicly sharing data or code —e.g. participant privacy or use of data from a third party—those must be specified.

Reviewer #1: Yes

Reviewer #2: Yes

Reviewer #3: Yes

**********

PLOS authors have the option to publish the peer review history of their article (what does this mean?). If published, this will include your full peer review and any attached files.

If you choose “no”, your identity will remain anonymous but your review may still be made public.

**Do you want your identity to be public for this peer review?**  For information about this choice, including consent withdrawal, please see our Privacy Policy.

Reviewer #1: No

Reviewer #2: No

Reviewer #3: No

# Editor report: Acceptance letter

**DOI:** 10.1371/journal.pcbi.1014772.r004

**Authors:** Dimitrios Vavylonis, Andreas Buttenschoen

PCOMPBIOL-D-26-00855R1

Mechanical polarity links adhesion-regulated protrusions to directional stability in glioblastoma cell migration

Dear Dr Sakumura,

I am pleased to inform you that your manuscript has been formally accepted for publication in PLOS Computational Biology. Your manuscript is now with our production department and you will be notified of the publication date in due course.

The corresponding author will soon be receiving a typeset proof for review, to ensure errors have not been introduced during production. Please review the PDF proof of your manuscript carefully, as this is the last chance to correct any errors. Please note that major changes, or those which affect the scientific understanding of the work, will likely cause delays to the publication date of your manuscript.

Soon after your final files are uploaded, unless you have opted out, the early version of your manuscript will be published online. The date of the early version will be your article's publication date. The final article will be published to the same URL, and all versions of the paper will be accessible to readers.

For Research, Software, and Methods articles, you will receive an invoice from PLOS for your publication fee after your manuscript has reached the completed accept phase. If you receive an email requesting payment before acceptance or for any other service, this may be a phishing scheme. Learn how to identify phishing emails and protect your accounts at https://explore.plos.org/phishing.

Thank you again for supporting PLOS Computational Biology and open-access publishing. We are looking forward to publishing your work!

With kind regards,

Janani Seenivasan

PLOS Computational Biology | Carlyle House, Carlyle Road, Cambridge CB4 3DN | United Kingdom ploscompbiol@plos.org | Phone +44 (0) 1223-442824 | ploscompbiol.org | @PLOSCompBiol
